# Doxycycline Alters the Porcine Renal Proteome and Degradome during Hypothermic Machine Perfusion

**DOI:** 10.3390/cimb44020039

**Published:** 2022-01-23

**Authors:** Leonie van Leeuwen, Leonie H. Venema, Raphael Heilig, Henri G. D. Leuvenink, Benedikt M. Kessler

**Affiliations:** 1 Department of Surgery, University Medical Center Groningen, University of Groningen, 9713 GZ Groningen, The Netherlands; l.h.venema@umcg.nl (L.H.V.); h.g.d.leuvenink@umcg.nl (H.G.D.L.); 2 Centre for Medicines Discovery, Nuffield Department of Medicine, Target Discovery Institute, University of Oxford, Oxford OX3 7FZ, UK; r.heilig@directbox.com (R.H.); benedikt.kessler@ndm.ox.ac.uk (B.M.K.); 3 Nuffield Department of Medicine, Chinese Academy of Medical Sciences Oxford Institute, University of Oxford, Oxford OX3 7FZ, UK

**Keywords:** renal transplantation, ischemia/reperfusion injury, machine perfusion, proteomics, degradomics

## Abstract

Ischemia-reperfusion injury (IRI) is a hallmark for tissue injury in donation after circulatory death (DCD) kidneys. The implementation of hypothermic machine perfusion (HMP) provides a platform for improved preservation of DCD kidneys. Doxycycline administration has shown protective effects during IRI. Therefore, we explored the impact of doxycycline on proteolytic degradation mechanisms and the urinary proteome of perfused kidney grafts. Porcine kidneys underwent 30 min of warm ischemia, 24 h of oxygenated HMP (control/doxycycline) and 240 min of ex vivo reperfusion. A proteomic analysis revealed distinctive clustering profiles between urine samples collected at T15 min and T240 min. High-efficiency undecanal-based N-termini (HUNTER) kidney tissue degradomics revealed significantly more proteolytic activity in the control group at T-10. At T240, significantly more proteolytic activity was observed in the doxycycline group, indicating that doxycycline alters protein degradation during HMP. In conclusion, doxycycline administration during HMP led to significant proteomic and proteolytic differences and protective effects by attenuating urinary NGAL levels. Ultimately, we unraveled metabolic, and complement and coagulation pathways that undergo alterations during machine perfusion and that could be targeted to attenuate IRI induced injury.

## 1. Introduction

Renal transplantation is the most effective treatment for patients suffering from end-stage renal disease [1]. Due to the shortage of donors, suboptimal kidneys, such as circulatory death donor (DCD) kidneys, are increasingly used to enlarge the donor pool [2,3,4]. Unfortunately, DCD kidneys are more likely to develop ischemia-reperfusion injury (IRI), which can result in short-term complications such as delayed graft function (DGF) or early graft failure [5,6]. IRI as consequence of a period of warm ischemia prior to organ retrieval is an important impeller of tissue injury in DCD kidneys. The pathogenesis of IRI is complex and involves acute hypoxia in the ischemic phase. Although reperfusion is essential for the reintroduction of oxygen, reperfusion itself causes additional injury due to massive mitochondrial production of reactive oxygen species (ROS), ATP depletion, cytoskeletal dysfunction and intracellular Ca^2+^ accumulation, which subsequently leads to activation of various injury pathways [7,8,9].

ROS production is not only detrimental to cells in many ways, it is an important modulator of protein function as well. It has been shown that high levels of ROS drive both matrix metalloprotease (MMP) expression and MMP activation [10]. More specifically, ROS induces the expression and activation of MMP-2 and MMP-9 [11,12,13,14], two proteases that play a prominent role in acute and chronic renal injury [15,16]. It has been shown that targeting MMP-9 greatly reduces renal tissue damage after IRI [14,17]. Doxycycline is a widely used antibiotic that, in addition to its anti-bacterial properties, inhibits MMP-9. MMP-9 exerts protective effects during renal IRI [18,19,20], however, the exact underlying mechanisms are still unclear.

As of 2016, static cold storage (SCS) has been replaced by hypothermic machine perfusion (HMP) as the standard of clinical care in The Netherlands for all kidneys donated by deceased donors. Multicenter randomized controlled trials have shown improved outcome in terms of a reduced incidence of DGF for HMP preserved kidneys compared to SCS [21,22]. Additionally, trials have shown that oxygenated HMP reduces post-transplant complications and significantly increases the one year graft survival rate compared to HMP without oxygen [23]. Besides improved preservation, HMP offers a therapeutic platform during organ preservation.

In this study, we explored the effect of doxycycline on proteolytic degradation mechanisms and urinary proteome of perfused kidney grafts. We performed an in-depth unbiased urinary proteomics and renal tissue degradomics analysis to assess whether doxycycline influences renal function at a molecular level during machine perfusion. Furthermore, we assessed renal function during ex vivo reperfusion, corroborating the altered metabolic, coagulation and complement pathways affected by doxycycline.

## 2. Materials and Methods

### 2.1. Experimental Design

Porcine kidneys (*n* = 7) were retrieved from a local abattoir and exposed to 30 min of warm ischemic time (WIT). Next, kidneys were preserved with oxygenated hypothermic machine perfusion for 24 h with (DOXY group) or without (control group) the addition of 100 µM doxycycline (Vibramycin SF 100 mg/5 mL, Pfizer BV, Capelle aan den Ijssel, The Netherlands) at a mean pressure of 25 mmHg at 4 °C using 100% oxygenated (100 mL/min) University of Wisconsin machine perfusion solution (Belzer MPS, Bridge to Life, Northbrook, IL, USA) and a Kidney Assist portable (XVIVO, Gothenburg, Zweden) perfusion machine. Currently the maximum HMP preservation time applied in the clinics in The Netherlands is 24 h. Thereafter, kidneys were reperfused in an ex vivo normothermic perfusion setup for 240 min with a mean arterial pressure of 80 mmHg at 37 °C using a custom-made perfusion machine. NMP specifications and analyses are shown in Appendix A Table A1. Samples were collected throughout HMP and reperfusion for renal function, proteomics and degradomics analysis.

### 2.2. Proteomic Analysis of Urine Using an In-Solution Digestion

To analyze the effect of doxycycline on the urinary proteome, urine samples collected at timepoint T15, T60, T120, T180 and T240 min of ex vivo reperfusion were concentrated and resuspended in 100 mM tetraethylammonium bromide (Merck, Darmstadt, Germany). Each sample was individually denatured and reduced using 5 mM dithiothreitol and alkylated using 20 mM iodoacetamide. Afterwards, all samples were cleaned up using a methanol-chloroform precipitation, and protein concentrations were determined using Pierce™ BCA Protein Assay Kit (Thermo Scientific, Waltham, MA, USA). 20 μg of protein per sample was digested using 0.4 μg of trypsin (Promega, Madison, WI, USA). Samples were acidified using trifluoroacetic acid, and cleaned up using the EVOSEP ONE (EVOSEP, Odense, Denmark) and Evotips following manufacturers instructions before high throughput label-free quantification using the timsTOF Pro (Bruker, MA, USA).

Raw data files were searched against a UniProt proteome database (Sus Scrofa, 2019) compiled from Uniprot using MaxQuant [24] with the following parameters: trypsin for enzyme specificity, allowing up to 2 missed cleavages and a minimum peptide length of 6, precursor mass tolerance at 10 ppm and fragment mass tolerance at 0.5 Da. Comprehensive analysis of the obtained data was performed using Perseus v1.6.7.0 [25]. Data was log2(x) transformed for normalization, and data was imputed by replacing missing data from normal distribution.

### 2.3. Tissue Degradome Analysis Using a TMT-HUNTER Workflow

To analyze protein degradation, snap frozen renal cortex tissue was collected before start of reperfusion (T-10) and after 240 min of reperfusion (T240) using a 23 mm needle biopsy gun (Invivo, Best, The Netherlands). Proteins were labelled using tandem mass tags (TMT) and prepared according to a high efficiency undecanal N-terminal enrichment (HUNTER) degradomics workflow as described by Weng et al. 2019 [26].

In short, tissue was homogenized in RIPA Lysis and Extraction Buffer (ThermoFisher) with 10% sodium dodecyl sulfate (SDS) and cOmplete™ Mini EDTA-free Protease Inhibitor Cocktail (Sigma, St. Louis, MO, USA) using Precellys 24 homogenizer (Bertin Instruments, Montigny-le-Bretonneux, France). Benzonase was added to degrade all DNA and RNA. Protein concentrations were determined using Pierce™ BCA Protein Assay Kit (Thermo Scientific). 150 µg of protein per sample was precipitated using acetone-methanol precipitation. Protein pellets were resuspended in 30 µL 6 M guanidine hydrochloride, 50 µL nanopure H_2_O, and 20 µL 1 M HEPES pH 8. Each sample was individually denatured and reduced using 10 mM tris(2-carboxyethyl)phosphine and alkylated using 25 mM iodoacetamide. Whole protein labelling at N-terminal α-amines site was performed using TMT 11-plex™ isobaric label reagents (Thermo Scientific). Labels were dissolved in anhydrous DMSO equal to sample volume and added to each sample and incubated for 1 h at room temperature. Samples were then quenched using 25 µL 1 M ethanolamine. Afterwards, all samples were combined creating three sample pools, cleaned up using an acetone-methanol precipitation, and digested using 30 µg of trypsin (Promega) per sample pool for 20 h. The combined sample was enriched for protein N-termini using 50:1 *w*/*w* undecanal and 40 mM sodium cyanoborohydride. Samples were acidified with 0.5% trifluoroacetic acid in 40% ethanol and the undecanal with bound tryptic peptides was removed using 0.1% trifluoroacetic acid in 40% ethanol and SOLA™ HRP Cartridges (Thermo Scientific). Samples were desalted using SOLA™ HRP Cartridges, dried down using vacuum centrifugation and resuspended in 0.1% trifluoroacetic acid. A nano-flow reversed phase chromatography-tandem mass spectrometry analysis (nLC-MS/MS) was performed using an Ultimate 3000 UPLC system coupled to a Q Exactive HF with mass spectrometer (Thermo Scientific) as described previously by Fye et al. [27]. In short, samples were separated on an EASY-Spray PepMap RSLC C18 column (500 mm × 75 mm, 2 mm particle size, Thermo Scientific) over a 60-min gradient of 2–35% acetonitrile in 5% DMSO, 0.1% formic acid at 250 nL/min.

Raw data files were searched against a UniProt proteome database (Sus Scrofa, 2019) compiled from Uniprot using Thermo Proteome Discoverer v2.3 software (Thermo Scientific). The following parameters were used: semi-trypsin for enzyme specificity, allowing up to 2 missed cleavages and a minimum peptide length of 6. Precursor mass tolerance at 10 ppm and fragment mass tolerance at 0.05 Da. Samples were normalized against corresponding pool. Comprehensional analysis was performed using Perseus v1.6.7.0 [25]. Data was log2(x) transformed, and data was imputed by replacing missing data from normal distribution.

### 2.4. Visualization and Statistics of Proteomics and Degradomics Data

Heatmaps were created by Z-scoring for normalization, and hierarchical clustering using Pearson correlation with average linkage for both row and column tree using Perseus. For visualizing the volcano plots, a two-sample T-test was performed using Perseus. GraphPad Prism 7.02 (GraphPad Software, San Diego, CA, USA) was used for plotting the log2 difference between the doxycycline group and the control group on the *x*-axis, and the −log10 (*p*-value) on the *y*-axis. The cut-off for statistical significance was set for −log10 (*p*-value) > 1.3. Pathway analysis and functional enrichment of proteins was performed using STRING 10.5 [28], PANTHER [29], and Cytoscape 3.8.2 [30]. TopFind [31] and Merops [32] were used for predicting and matching proteolytic events to most probable responsible protease for peptides searched against a human proteome database (Uniprot, Geneva, Switzerland, 2021) as sus scrofa is not supported yet.

## 3. Results

### 3.1. Kidney Urine Proteomics and Tissue Degradomics Profiles

**Figure 1:**
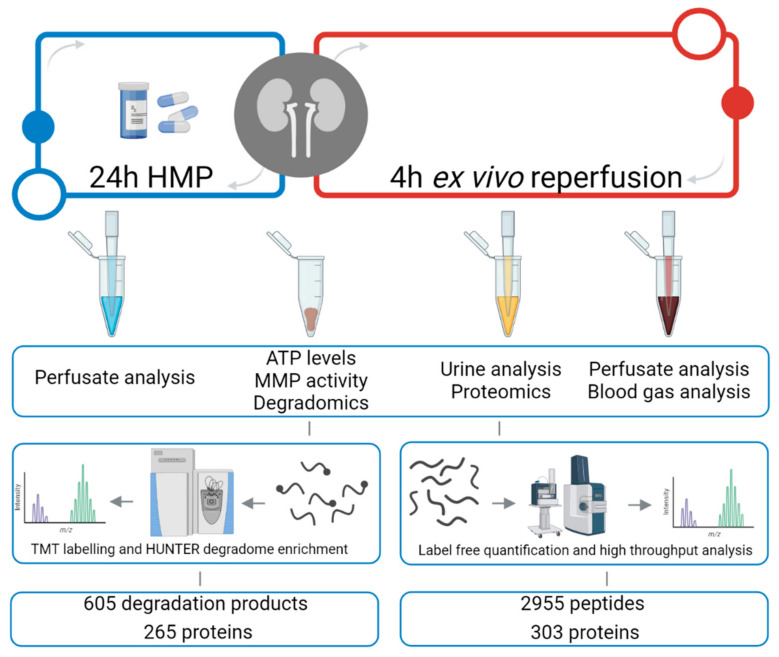
Urine proteomics & kidney tissue degradomics experimental workflows. Porcine kidneys (*n* = 7 per group) were retrieved from a local abattoir and exposed to 30 min of warm ischemia, 24 h of oxygenated hypothermic machine perfusion (HMP) with or without the addition of doxycycline, and 4 h of ex vivo reperfusion for functionality assessment. Perfusate, tissue and urine samples were collected during HMP and ex vivo reperfusion for various analyses. Protein degradation was analyzed using renal cortex tissue collected before and after ex vivo reperfusion and using a HUNTER [26] degradomics workflow. Urinary proteomics was performed on collected ultra-filtrate during ex vivo reperfusion using a label-free quantitative proteomics workflow. Illustration is original and created using www.biorender.com (accessed on January 2022).

The experimental workflow is shown in Figure 1. We first analyzed the urinary proteome. Global analysis resulted in 2955 identified peptides that were matched to 303 unique proteins at a 1% false discovery rate (FDR).

Next, we analyzed the kidney tissue degradome using high-efficiency undecanal-based N-termini enrichment (HUNTER) [26]. Global analysis resulted in 717 identified peptides, potentially reflecting freshly cleaved N-termini of degradation products. 605 peptides were quantifiable due to labelled N-terminal α-amines. This means that after N-terminome enrichment, 85% of the sample represented the quantifiable degradome. These 605 degradation products belong to 265 unique proteins.

### 3.2. Doxycycline Causes Molecular Alterations in Urine during Machine Perfusion

**Figure 2:**
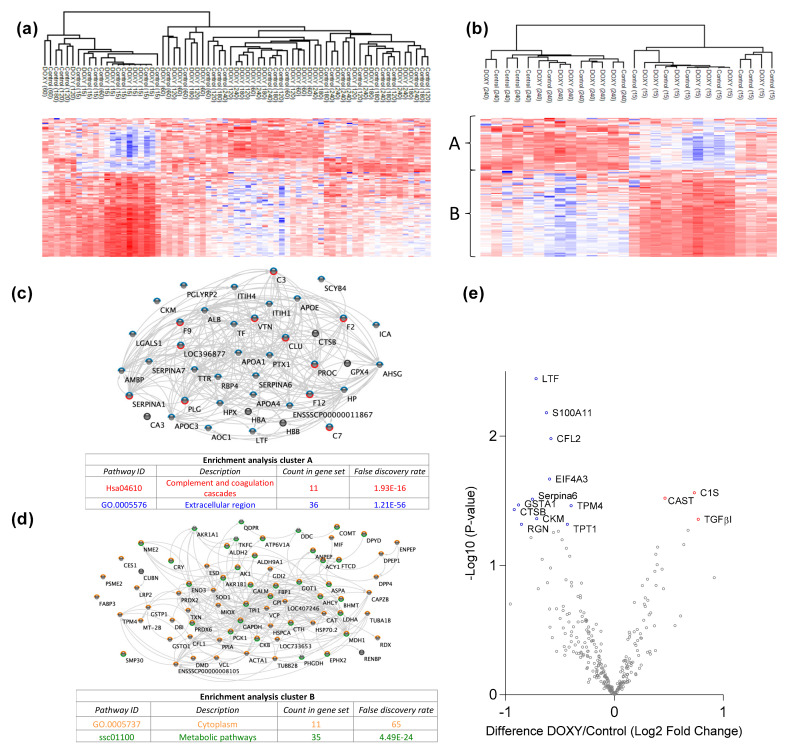
Urinary proteomic analysis of ex vivo reperfused kidneys revealed altered complement & coagulation, ECM and metabolic pathways. (**a**) Heat map and hierarchical clustering of proteins at T15, T60, T120, T180 and T240. (**b**) Heat map and hierarchical clustering of proteins in urine samples only at T15 (beginning) and T240 (end). Protein expression is clustered into A and B. (**c**) Gene ontology term enrichment analysis of proteins in cluster A. (**d**) Gene ontology term enrichment analysis of proteins in cluster B. (**e**) log2 Fold Change in abundance between DOXY and the control was plotted on the *x*-axis and the statistical significance of these changes on the *y*-axis as the −log10 (*p*-value). DOXY: doxycycline treated.

We first analyzed the urinary proteome. Pathway analysis of the 303 identified proteins showed that most of these proteins belonged to metabolic pathways and complement and coagulation cascades. Unsupervised clustering was performed to examine data uniformity. No clustering was observed between the treatment groups, however clustering of urine samples taken at time point 15 min was observed (Figure 2a). Expression profiling showed that the addition of doxycycline caused significantly more downregulation of proteins in the urine compared to control at T120, T180 and T240 min.

To analyze whether the urinary proteome differs between the beginning and the end of kidney reperfusion, unsupervised clustering using urine samples of T15 and T240 was performed. Distinctive clustering was observed between the two timepoints (Figure 2b). Based on hierarchical clustering of the protein expression, two row clusters were identified. Cluster A consists of 34 proteins that are mostly located in extracellular regions and proteins that play a role in complement and coagulation pathways (Figure 2c). These proteins were upregulated at T240. Cluster B consists of 72 proteins that are mostly located in the cytoplasm and play a role in metabolic pathways (Figure 2d). These proteins were upregulated at T15. Figure 2e and Table S1 show significant differentially expressed proteins in the urine between the two experimental groups. C1s, TGF-β1, and Calpastatin were significantly more secreted into the urine of kidneys treated with doxycycline.

### 3.3. Doxycycline Alters Renal Degradome

**Figure 3:**
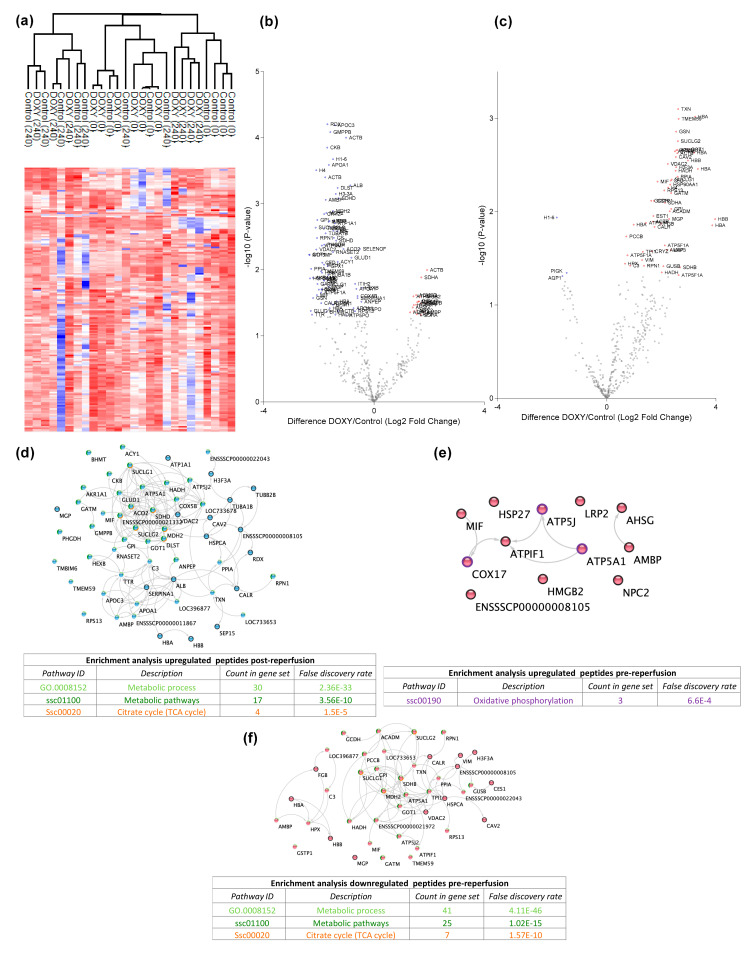
Renal tissue degradomics during ex vivo reperfusion modulated by doxycycline. (**a**) Heat map and hierarchical clustering of degradation products across all tissue samples. (**b**) Represents the log2 fold change of degradation products between DOXY and the control group pre-reperfusion (T-10). (**c**) Represents the log2 fold change of degradation products between DOXY and the control group post-reperfusion (T240). (**d**) Represents a KEGG pathways analysis of degradation products identified as significantly downregulated and (**e**) upregulated pre-reperfusion. (**f**) Represents a KEGG pathways analysis of degradation products identified as significantly upregulated post-reperfusion. DOXY; doxycycline treated.

Next, we analyzed the kidney tissue degradome. Unsupervised clustering of identified degradation products was performed to examine data uniformity. No clustering was observed between experimental groups (Figure 3a). Figure 3b shows the degradation products significantly up- and downregulated in the doxycycline group compared to control (Table S2). At T-10, more degradation products were observed in the control group. At T240, more degradation products were observed in the doxycycline group (Figure 3c; Table S3).

At T-10, 72 significant degradation products were identified in the control group (Figure 3d). String pathway analysis showed that the majority of these proteins belonged to metabolic processes. A total of 19 significant degradation products were identified in the doxycycline group (Figure 3e). String pathway analysis showed that the majority of these proteins belonged to oxidative phosphorylation.

At T240, 3 significant degradation products were identified in the control group, namely GPI-anchor transamidase, histone H1t and aquaporin-1 (Figure 3c). 51 significant degradation products were identified in the doxycycline group (Figure 3f). String pathway analysis showed that the majority of these proteins belonged to metabolic processes.

### 3.4. Protease Activity in Experimental Groups

PANTHER protein class analysis of both groups led to the identification of 20 proteases. These proteases belong to cysteine, metallo and serine proteases (Table 1). Using a human database in combination with TopFind [31] analysis, responsible proteases for the identified substrates in each sample were predicted. At T-10, 17 substrates could be linked to the responsible protease in the doxycycline group and 15 substrates in the control group (Table 2). At T240, 2 substrates could be linked to the responsible protease in the doxycycline group (Table 2). No predictions could be made for the control group at T240.

### 3.5. Protein Degradation during Ex Vivo Reperfusion

**Figure 4:**
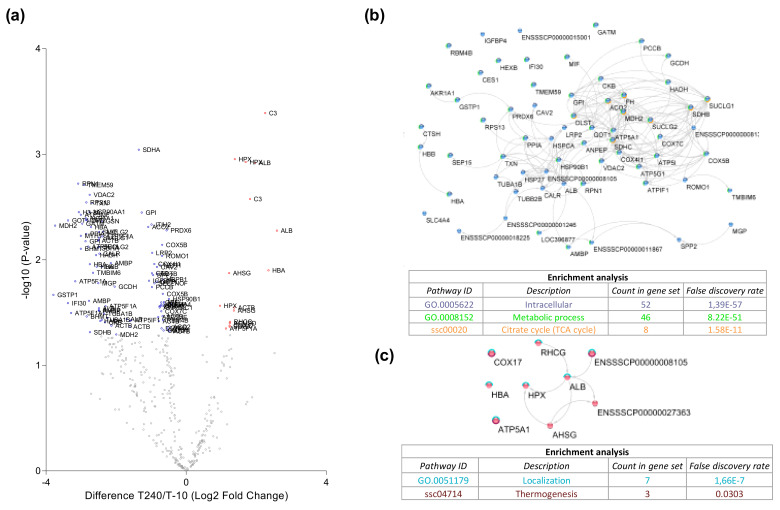
Kidney degradome dynamics during ex vivo reperfusion. (**a**) Log2 Fold Change in abundance between post (T240) and pre (T-10) reperfusion was plotted on the *x*-axis and the statistical significance of these changes on the *y*-axis as the -log10 (*p*-value). Significantly downregulated degradation products (blue). Significantly upregulated degradation products (red). (**b**) String cluster and KEGG pathway analysis of degradation products significantly downregulated after reperfusion. (**c**) String clustering and KEGG pathway analysis of degradation products significantly upregulated after reperfusion.

To characterize the effect of ex vivo reperfusion, the significant differences in degradome pre- and post-reperfusion were analyzed in just the control group. Significantly more proteins were degraded after HMP (T-10) compared to after reperfusion (T240) (Figure 4a). At T-10, 69 degradation products were significantly more abundant. String pathway analysis showed that the majority of these products were intracellular (Figure 4b). At T240, 16 degradation products were significantly more abundant. String pathway analysis showed that the majority of these products were localization proteins (Figure 4c).

### 3.6. Renal Function and Injury Markers during HMP and Reperfusion

**Figure 5:**
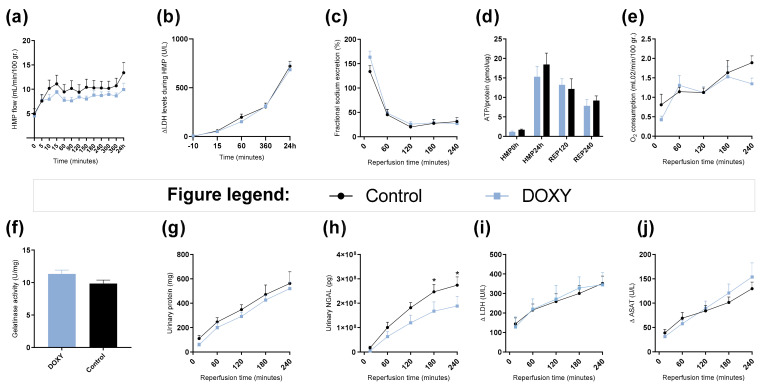
Renal function during hypothermic machine perfusion and ex vivo reperfusion. (**a**) Arterial flow during hypothermic machine perfusion (HMP) shown in mL/min/100 gr. of kidney weight over time. (**b**) LDH levels in perfusate in U/L during HMP. (**c**) Fractional sodium excretion shown in % during reperfusion. (**d**) ATP levels in cortex tissue shown in pmol per ug protein at time point: HMP0h, HMP24h or pre-reperfusion, 120 min into reperfusion and 240 min into reperfusion or post-reperfusion. (**e**) Oxygen consumption shown in mL O_2_/min/100 gr. of kidney weight during reperfusion. (**f**) Gelatinase activity after 240 min of reperfusion. (**g**) Absolute accumulative urinary protein levels in mg. (**h**) Absolute accumulative urinary NGAL levels in pg. (**i**) delta LDH levels in perfusate in U/L during reperfusion. (**j**) delta ASAT levels in perfusate in U/L during reperfusion. * = *p* < 0.05. Data shown as mean ± SEM. DOXY; doxycycline treated.

There were no significant differences observed in renal function during HMP (Figure 5). During reperfusion, urinary neutrophil gelatinase–associated lipocalin (NGAL) levels were significantly lower in doxycycline group at T180 min (*p* = 0.0430) and T240 min (*p* = 0.0297) compared to control (Figure 5h).

## 4. Discussion

Ischemia-reperfusion injury in renal transplantation is detrimental for the recipient as it can lead to short- and long-term graft failure [5,33]. Unfortunately, research into targeting IRI as well as identifying novel drug targets is limited. Omics technologies are used for discovering specific molecular processes that play a role in disease [34]. The goal of proteomics is to acquire a more global overview by examining as many proteins of a cell as possible, instead of each one individually [35]. Proteases such as the MMPs are enzymes that cause protein degradation and therefore play essential roles in biological processes and pathological implications [36]. Determining the degradome and responsible proteases can provide molecular clues about the functionality of a biological pathway. In addition, proteases are druggable targets, thereby offering pharmacological intervention strategies to modulate proteolytic pathways [37,38]. However, generally, endogenous expression levels of proteases and protease inhibitors are low, which makes them challenging to detect efficiently. Weng et al., 2019 developed a high-efficiency undecanal-based N-termini enrichment (HUNTER) method for degradome analysis of microscale samples [26]. HUNTER allows mapping of the exact location of the biologically generated N-termini, generated through the cleavage of a peptide bond by the protease that is responsible for this proteolytic activity. Proteomic profiling with the use of mass spectrometry has been widely adapted in medical research including the field of kidney failure and transplantation [39,40]. Our goal was to observe the effect of doxycycline addition during HMP on renal proteome and degradome during ex vivo normothermic machine perfusion. Furthermore, we explored the general effect of machine perfusion on the renal degradome.

### 4.1. Protein Secretion during Ex Vivo Reperfusion

Proteinuria is a common phenomenon after renal transplantation and is correlated with post-transplant graft survival [41]. Rising urinary protein levels were also observed during ex vivo reperfusion (Figure 5). To our knowledge, urine collected during ex vivo reperfusion has never been thoroughly analyzed. As urine is the direct ultra- filtrate of the kidneys especially in an ex vivo setting, it provides accurate information on kidney functionality. Therefore, we analyzed the effect of doxycycline on urinary proteome. Furthermore, we analyzed the urinary proteome during ex vivo reperfusion. When globally observing the urinary proteome, no clustering occurred between groups. However, when looking per time point, significantly more proteins were secreted in the control group.

### 4.2. Changes in Metabolic Pathways

Interestingly, the urinary proteome of both groups at T15 consisted mostly of proteins belonging to metabolic pathways. Renal cells require high levels of energy due to their ATP-dependent functions like reabsorption and secretion [42]. Hypoxic injury during ischemia causes production of ROS [43]. Excessive ROS production alters the cellular redox potential and causes impairment in electron transport, energy metabolism and causes ATP depletion. Many of the identified degradation products belong to metabolic pathways as well, indicating that metabolism is disturbed during IRI. Treatment that protects metabolic pathways should therefore be administrated at an early stage of ex vivo machine perfusion.

### 4.3. Changes in Complement and Coagulation Cascades

The urinary proteome at T240 of both groups consisted of many proteins belonging to complement and coagulation cascades. It has been shown that the complement system is associated with the inflammatory response to IRI [44,45]. IRI causes the release of danger-associated molecular patterns (DAMPs), neo-antigen formation, and immune complex formation that then can activate the complement system by any of the three complement pathways [46]. Even though the underlying mechanisms are still unclear, complement activation due to IRI contributes to the pathogenesis of renal fibrosis. Significant upregulation of C1s secretion in the urine of the doxycycline group was observed. This could indicate that there is detachment of remnant classical complement attack complexes in the doxycycline group. Interestingly, our group has shown that upregulation of C1s secretion during hypothermic machine perfusion correlates with a good kidney function 1-year post transplantation [47]. Furthermore, we identified significant downregulation of C3 degradation products before reperfusion and significant upregulation C3 degradation products after reperfusion (Figure 3b,c). It has been shown that C3 formation, and degradation after complement activation by all three pathways plays a role in renal ischemic injury [48,49]. Inhibition of complement activation could provide novel therapeutic targets to ameliorate fibrosis during ex vivo reperfusion.

### 4.4. Protein Degradation during Ex Vivo Reperfusion

Protein degradation plays an important role in physiological functioning of the kidney [50,51]. Excessive protein degradation can lead to loss of tissue integrity and subsequent loss of renal function. However, lack of protein degradation can lead to the accumulation of proteins and fibrosis formation which in turn can also lead to loss of renal function. The delicate balance between synthesis and degradation is therefore key for renal function. To our knowledge, no studies have focused on protein degradation in a renal transplant setting. We therefore studied protein degradation in our controlled experimental setup. More degradation products were observed in the control group after HMP. These results indicate that the addition of doxycycline possibly attenuates protein degradation during HMP. Additionally, more degradation products were observed after hypothermic compared to normothermic perfusion. This indicates that degradation products are not all flushed out during HMP and that they are most likely recycled during ex vivo reperfusion for tissue repair.

### 4.5. Proteases as Possible Molecular Targets

The protease class that was most active in both groups were cysteine proteases. Both calpain-1 and 2 were identified during ex vivo reperfusion (Table 1). Calpains play a role in Ca^2+^ signaling and are activated by accumulated intracellular Ca^2+^ during ischemia [9,52]. Activated calpain-1 and 2 selectively cleave cytoskeletal and membrane proteins [53]. We observed many degradation products of cytoskeletal proteins after HMP (Figure 4), possibly caused by activated calpains. Furthermore, we identified significantly more urinary secretion of calpastatin in the doxycycline group. Calpastatin is an endogenous inhibitor protein that targets the Calpains [54]. Previous studies have shown that calpastatin is downregulated in heart and brain tissue after ischemia-reperfusion injury [55,56]. Overexpression of calpastatin and thus secretion could potentially have a protective effect against IRI; and targeting calpains during NMP could possibly protect the kidney against ischemia [57,58].

TopFind analysis predicted that the identified actin and tubulin substrates were most probably cleaved by Serine protease HTRA2, granzyme M, cathepsin S and caspase 3 (Table 2). Interestingly, research has shown that proteolysis of calpastatin by caspase 3 may regulate calpain activity during IRI [59], suggesting interplay between these proteases, calpastatin and cytoskeletal integrity. The prediction was based on a human database so validation studies need to be performed to identify the exact responsible proteases.

Meprin α/β metalloproteinases were predicted to be active in both groups. They are extracellular proteases that are involved in connective tissue homeostasis, intestinal barrier function and immunological processes. Dysregulation is associated with acute kidney injury, chronic inflammation and fibrosis [60,61]. Targeting meprin α/β metalloproteinases during machine perfusion could therefore potentially benefit the kidney.

### 4.6. Effect of Doxycycline

The addition of doxycycline during HMP did not affect renal function during HMP (Figure 5a,b). Doxycycline did result in significant attenuation of urinary NGAL levels during ex vivo reperfusion (Figure 5h). NGAL is a protein covalently bound to gelatinase from neutrophils [62]. It is an acute tubular damage marker after ischemia, and an early fibrosis marker with a great predictive value of post-transplant function [63,64,65,66], indicating that doxycycline does potentially have renal protective effects during HMP. These results are in line with Moser et al. 2016. [67]. They showed that the addition of doxycycline under hypothermic conditions has protective effects on rat kidneys as it attenuated injury markers such as secretion of NGAL and reduction of MMP-2 and MMP-9 activity. Furthermore, Cortes et al. 2018 showed that doxycycline treatment attenuated renal injury, and downregulated MMP-2 and -9 levels in rat kidneys [20]. We did not see the same effects on gelatinase inhibition in our porcine kidneys (Figure 5f). This could be due to interspecies differences, or the dose-response and thermodynamics of doxycycline under hypothermic conditions. Furthermore, the enzymatic activity of the MMPs is significantly influenced by temperature [68]. As normothermic machine perfusion is now increasingly used, it would be beneficial to analyze whether doxycycline shows more prominent effects under normothermic conditions when the kidney graft is 100% metabolically active [42].

### 4.7. Clinical Implementation

Although further studies are needed to confirm whether the addition of doxycycline is beneficial during machine perfusion, the emergence of machine perfusion itself provides opportunities for marginal graft repair by means of ex vivo targeted drug delivery [69]. We have identified multiple pathways such as metabolic pathways and complement and coagulation pathways that show upregulated degradation during NMP. Furthermore, we have identified several relevant proteases that would make interesting drug targets during NMP. Ultimately, this novel approach will lead to better short and long-term graft function and improve the patient’s quality of life.

### 4.8. Limitations

We were not able to analyze the effects of doxycycline beyond NMP, as these kidneys were obtained from an abattoir and thus transplantation was impossible. However, our model has shown to be a valid technique for assessment of kidney quality during reperfusion [70,71,72,73,74,75]. Kidneys obtained from waste material provide a great platform to test early hypotheses without the need of laboratory animals, thereby reducing both costs and animals for research.

Unfortunately, many cleavage sites within the proteome are still unidentified, and we could only match a handful of substrates to their possible responsible protease. Especially for proteolytic activity in porcine samples, as the prediction software is currently not compatible for the sus scrofa degradome. It would be interesting to unravel all proteases responsible for the identified proteolytic activity. Furthermore, it would be valuable to validate these predictions by spiking the protein of interest with the protease of interest. This way we can see if this protease is really responsible for the identified proteolytic activity.

In addition, this pilot study consisted of a small sample size. Validation using larger groups will be needed to further unravel the underlying pathophysiological pathways of IRI.

## 5. Conclusions

In conclusion, the addition of doxycycline during HMP led to significant proteomic differences. We unraveled multiple pathways that undergo alterations during machine perfusion. Pathways that could be targeted using ex vivo machine perfusion to attenuate IRI induced injury. Furthermore, we showed that doxycycline administration during HMP shows protective effects by attenuating urinary NGAL levels. More validation studies need to be conducted before implementing doxycycline supplementation during machine perfusion preservation.

## Figures and Tables

**Table 1:** Identified proteases in both sample groups using Panther pathway analysis.

Cysteine Proteases	Metallo Proteases	Serine Proteases
CTSB	FOLH1	PLG
USP37	ANPEP	APEH
CTSH	MMP1	DPP4
CAPN1	THOP1	CFD
CAPN2	NLN	HP
UCHL3	ENPEP	ESD
CTSL		
UCHL1		

**Table 2:** Proteases identified in experimental groups and their proteolytic activity.

Doxycycline T-10		Control T-10	
**Accession Number** **Gene Name**	**Meprin α/β Metalloproteinase** **Cleavage Sites**	**Accession Number** **Gene Name**	**Meprin α/β Metalloproteinase** **Cleavage Sites**
P10809 HSPD1	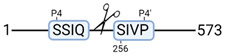	P10809 HSPD1	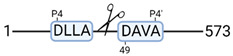
Q99497 PARK7	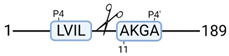	P37802 TAGLN2	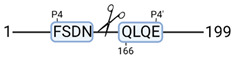
Q15651 HMGN	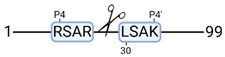	P11142 HSPA8	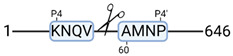
P22626 HNRNPA2B1	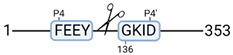	P08670 VIM	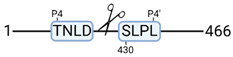
**Accession number** **Gene name**	**Serine protease HTRA2** **Cleavage sites**	P06396 GSN	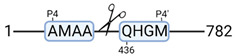
P68371 TUBB4B	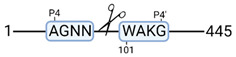	P14866 HNRNPL	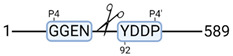
P63267 ACTG2	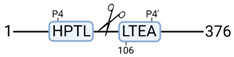	**Accession number** **Gene name**	**Serine protease HTRA2** **cleavage sites**
P68366 TUBA4A	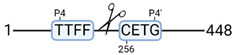	P68363 TUBA1B	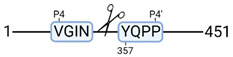
**Accession number** **Gene name**	**Cathepsin S** **cleavage sites**	P68371 TUBB4B	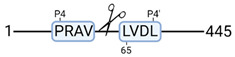
P60709 ACTB	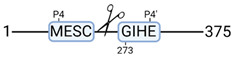	**Accession number** **Gene name**	**Caspase 3** **cleavage sites**
O60749	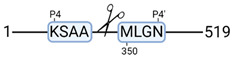	P63267 ACTG2	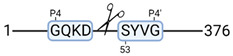
**Accession number** **Gene name**	**Granzyme M** **cleavage sites**	**Accession number** **Gene name**	**Granzyme M** **cleavage sites**
O75367 H2AFY	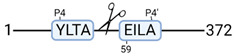	P16402 HIST1H1D	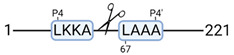
P16402 HIST1H1D	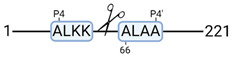	P16402 HIST1H1D	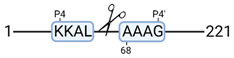
P60709 ACTB	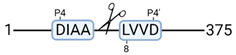	P16402	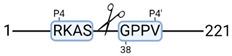
**Accession number** **Gene name**	**Cathepsin B** **cleavage sites**	**Accession number** **Gene name**	**Granzyme B** **cleavage sites**
P52272 HNRNPM	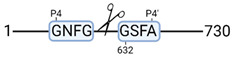	P68104 EEF1A1	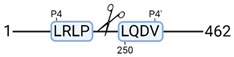
**Accession number** **Gene name**	**Mitochondrial-processing peptidase β cleavage sites**	**Accession number** **Gene name**	**Cathepsin D** **cleavage sites**
Q99643 SDHC	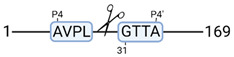	P68871 HBB	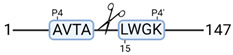
**Accession number** **Gene name**	**Tripeptidyl-peptidase 1** **cleavage sites**		
O14773 TPP1	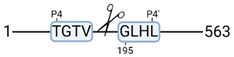	**Doxycycline T240**	
**Accession number** **Gene name**	**Cathepsin L1** **cleavage sites**	**Accession number** **Gene name**	**Meprin α/β metalloproteinase** **cleavage sites**
P53634 CTSC	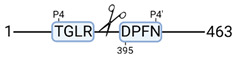	P14866 HNRNPL	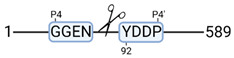
**Accession number** **Gene name**	**Matrix metalloprotease 11** **cleavage sites**	**Accession number** **Gene name**	**Cathepsin S** **cleavage sites**
P62937	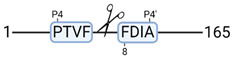	O00193 SMAP	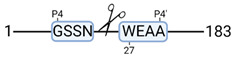

## Data Availability

The mass spectrometry proteomics data have been deposited to the ProteomeXchange Consortium (http://proteomecentral.proteomexchange.org (accessed on: 30 November 2021)) via the PRIDE partner repository [76] with the dataset identifier PXD029599.

## Supplementary Materials

The following are available online at https://www.mdpi.com/article/10.3390/cimb44020039/s1, Table S1. Identified proteins in the urine during NMP; Table S2. Identified degradation products pre-reperfusion (T-10). Table S3. Identified degradation products post-reperfusion (T240).

## Appendix A

**Table A1:** NMP specifications.

**Perfusate Composition:**		
Heparinized and leukocyte depleted autologous blood		500 mL
Ringers lactate solution (Baxter)		280 mL
Amoxicillin/Clavulanic acid (1000 mg/200 mg)		1 ampule
8.4% Sodium Bicarbonate (B. Braun)		10 mL
5% glucose (Baxter)		10 mL
Dexamethasone (20 mg/mL) (Centrafarm)		333 μL
Mannitol (Merck)		6 mg
Creatinine (Merck)		90 mg
Sodium Nitroprusside (Merck)		2 mg
**Infusion solution: 20 mL/h**		
Aminoplasmal (B. Braun)		90 mL
8.4% Sodium Bicarbonate		2.75 mL
Insulin (100 IU/mL) (NovoRapid)		0.186 mL
**Corrections based on blood gas values:**		
If glucose < 8 mmol/L, 5% glucose administrated to a concentration of 8 mmol/L.		
If pH under 7.3, 8.4% sodium bicarbonate administrated using Henderson–Hasselbalch equation to obtain a pH of 7.35.		
**Renal function analysis:**		
Fractional sodium excretion	Creatinine and sodium concentrations were determined in perfusate samples and corresponding urine samples, in a routine fashion at the lab of clinical chemistry (UMCG). Calculated as: (urinary sodium levels × urinary flow)/(creatinine clearance × perfusate sodium levels).	
Oxygen consumption	Calculated using pO_2_ differences between arterial and venous sites, measured with a pH-blood gas analyzer ABL90 FLEX (Radiometer, Brønshøj, Denmark) and expressed as mL O_2_/min/100 r. according to trans-renal flow and kidney mass pre-reperfusion using the equation as described by Venema et al. 2019 [77].	
ATP/Protein	Tissue samples collected after 30 min of warm ischemia, 24 h of HMP, 120 min of reperfusion and 240 min of reperfusion were stored in sonification solution (0.372 g EDTA in 130 mL H_2_O and NaOH, pH 10.9 + 370 mL 96% ethanol). A bioluminescence kit was used (Roche Diagnostics). Luminescence was measured using a luminometer (Packerd LumiCountä, IL, USA). The obtained ATP value was normalized to the total protein content using Pierce^TM^ BCA protein assay kit. The final ATP content was expressed as pmol ATP/μg protein.	
LDH & ASAT	Concentrations were measured in HMP and NMP perfusate in a routine fashion at the lab of clinical chemistry (UMCG).	
Urinary protein concentrations	Determined using Pierce™ BCA Protein Assay Kit (Thermo Scientific) according to manufacturer’s instructions.	
Urinary NGAL	Determined using an ELISA kit (Eurobio, Les Ulis, France) according to manufacturer’s instructions.	
MMP activity	Measured using the Gelatinase (Gelatin Degradation/Zymography) Assay Kit (BioVision, San Francisco, CA, USA) according to manufacturer’s instructions.	
Statistics	GraphPad Prism 7.02 (GraphPad Software, USA) was used for visualizing data and statistical analyses. Values are shown as means with corresponding standard error of the mean. Continuous variables were plotted over time. Statistical differences between groups for each timepoint were determined using 2 way ANOVA and Fisher’s LSD for multiple comparisons. The cut-off for statistical significance was set at *p* < 0.5.	
