# Supplementary material for: Doxycycline Alters the Porcine Renal Proteome and Degradome during Hypothermic Machine Perfusion
Source: Curr Issues Mol Biol. 2022 Jan 23;44(2):559–77. doi: 10.3390/cimb44020039 (PMC8928973; doi:10.3390/cimb44020039)
Supplement: Supplementary file 1 [file cimb-44-00039-s001.zip › Table S1.pdf]

Table S1. Identified proteins in the urine during NMP

Accession number	Difference (DOXY vs Control)	-Log10 (P-value)
P14632	-0,718323538	**2,443344056**
Q06AA3	-0,853458415	**1,317865238**
P51781	-0,880076761	**1,467952651**
P61288	-0,432462151	**1,318433906**
Q9GK37	-0,753159046	**1,511834839**
Q5G6V9	-0,583474712	**1,980557928**
P67937	-0,397668592	**1,462279948**
Q5XLD3	-0,713990797	**1,362004962**
A1E295	-0,921003576	**1,431082964**
A6M931	-0,595868215	**1,667335577**
P31950	-0,62466285	**2,180157401**
P12675	0,465654921	**1,520396018**
Q69DK8	0,735955609	**1,562074673**
O11780	0,771671426	**1,357322297**
Q29052	0,094118907	0,26559026
Q29268	0,082456301	0,138208932
Q29068	-0,529800827	0,336133319
Q29261	0,121866841	0,103343556
Q29092	-0,09303285	0,093728667
Q29073	-0,329194749	0,408525668
Q29236	0,638073691	1,161634829
Q29228	-0,035598575	0,041960405
Q29214	0,43823039	0,971511384
Q29099	0,18938702	0,39588393
Q29243	-0,066607557	0,129614095
Q29221	0,238803367	0,472385818
Q29371	-0,195320761	0,506843136
Q29318	-0,181405615	0,139461341
Q2HYU2	-0,084957314	0,160768472
Q2EN76	0,326246659	0,335782153
Q2EN75	-0,084926831	0,138502435
Q29594	-0,005294279	0,009051013
Q29561	-0,061972116	0,065187845
Q29550	-0,614821512	0,866379692
Q29315	0,307664646	0,730534804
Q29549	-0,166064814	0,544962967
Q29545	0,027828042	0,08563466
Q29387	-0,065920829	0,10514791
Q29384	0,222791269	0,304389744
Q29375	0,198641051	0,381582371
Q29048	-0,288357765	0,301359592
Q29361	-0,240697265	0,487138905
Q29548	-0,192797844	0,419545411
Q29043	-0,9552374	0,702432462
Q28944	-0,237113529	0,096641251
Q29024	-0,152450591	0,267138169
Q02110	0,120570149	0,115351628
Q02038	-0,028203174	0,038221954

Q007T2	-0,395366026	0,456585213
P82460	-0,532224624	0,665162982
P82125	0,043239486	0,044438247
P81608	0,211131171	0,503970117
Q03472	-0,141093503	0,299976836
P81405	-0,168361753	0,333076814
P80895	0,043178013	0,051742947
P80276	-0,081600579	0,084213092
P80230	-0,012959564	0,021102256
P80229	-0,058810706	0,066331191
P80041	0,118419461	0,156400714
P80031	0,084050961	0,14281615
P80928	-0,595652157	0,662833081
Q03710	-0,024854984	0,037526154
Q06AB3	0,137714814	0,277008608
Q06AU6	-0,026872054	0,056051437
Q28960	-0,073125365	0,064433821
Q2XQV4	-0,020322937	0,026152719
Q28943	0,012398551	0,00810174
Q28833	0,451510578	0,819697294
Q1KYT0	-0,063734138	0,122670968
Q19S50	0,071300212	0,042302403
Q19QT7	0,245995355	0,229464277
Q19PY3	-0,233410281	0,471815927
Q19KI0	-0,125080201	0,163265926
Q19AZ8	0,049380381	0,105207079
Q0MVN8	-0,245804604	0,294621531
Q08094	-0,207401092	0,454938485
Q08092	-0,394004104	0,868770135
Q07717	-0,279086118	0,63331024
Q06AU7	-0,138326356	0,255314665
Q29041	-0,345293136	0,443364381
A0PFK7	0,010380641	0,008924128
Q49I35	-0,290964341	0,367832847
Q2XVP4	-0,042964105	0,030465848
Q95334	-0,252972065	0,202178026
Q95333	-0,122215041	0,104063767
Q95332	-0,158978526	0,189899806
Q95281	0,275938101	0,700249889
Q95276	-0,056693781	0,11095739
Q95250	-0,165792928	0,364882915
Q95342	0,30423182	0,893024793
Q8WN98	-0,391825155	0,486367901
Q8SPS7	0,368898374	0,532524674
Q8SPK0	0,056026491	0,092009916
Q8MJ30	-0,067491079	0,058010118
Q8MJ14	-0,662881188	1,048080281
Q8MIR4	-0,441336572	1,132310003
Q8MIB3	-0,312206985	0,749467465
Q8SQ26	-0,036623161	0,033758794

Q95ND5	-0,066642553	0,132404273
Q99028	0,040360444	0,060560231
Q9BDJ5	0,270431041	0,576800177
Q9TV62	-0,057181333	0,085119937
Q9TV61	-0,498141437	0,589966509
Q9TUQ3	0,125163657	0,42775581
Q9TT35	0,111506052	0,140478919
Q9TSX9	-0,083610107	0,148094616
Q9TRC7	-0,017767635	0,025434923
Q9N1F5	-0,283623441	0,310292864
Q9MYY8	-0,248528929	0,304149222
Q9GMB0	0,122557654	0,222204891
Q9GLP2	0,108702192	0,294193047
Q9GLP1	0,91919245	0,905936562
Q9GL51	-0,226482728	0,121052364
Q9GKX6	0,001240445	0,001649877
Q9GJT2	-0,160830813	0,181373047
Q9BEC7	-0,699364796	0,819802203
Q8HZJ6	0,393831564	1,005205499
Q866Y3	0,103752309	0,267072735
Q863Z0	-0,569674199	0,442402263
Q7YQE5	0,089920296	0,162559836
Q5S1S4	-0,376967188	0,526022082
Q5PYH3	0,115666254	0,215640333
Q5ISC6	-0,145443956	0,224268295
Q5GN48	-0,167697068	0,210696027
Q58D68	0,065849473	0,090309337
Q52NJ6	-0,131962609	0,27112134
Q52NJ4	-0,120010691	0,235827712
Q52NJ2	-0,149362554	0,148061085
Q52NJ1	-0,017356538	0,024276837
Q52I78	-0,008693617	0,013107428
Q4JHS0	0,397147856	1,14091863
Q4GWZ2	-0,029940933	0,018699452
P80015	-0,303134144	0,863058124
Q3ZD69	0,156748274	0,244896125
Q2YGT9	0,110994956	0,206896687
Q5S1U1	-0,163275485	0,131379514
Q2XSV9	0,078111624	0,100547314
Q64L94	0,383104314	0,846233399
Q6PQZ1	-0,158304553	0,145242465
Q7SIB7	0,011438317	0,015691704
Q7PCJ9	-0,050149489	0,044797558
Q7M2W6	0,284094062	0,682331446
Q767L7	0,154510034	0,174804786
Q767L6	-0,513783574	1,264360491
Q764M5	-0,255754317	0,368368595
Q711S8	0,14471159	0,249714514
Q710C4	-0,083462762	0,069013052
Q6S4N2	-0,303953306	0,322202333

Q6RI85	-0,208492875	0,414800306
Q6QAT1	-0,231966679	0,484855167
Q6QAT0	-0,333617583	0,760763201
Q6QAQ1	-0,030365322	0,061601235
Q6Q7J2	-0,053335042	0,071415405
Q6Q2C2	0,197491235	0,154499546
Q69DL0	0,284585955	0,749129427
P79381	0,122284526	0,097471896
P79324	-0,084755095	0,146816023
P79379	-0,196410059	0,182729697
P04163	-0,116771268	0,169145311
P03974	-0,025068709	0,017136812
P02554	0,139057378	0,107134768
P02543	-0,558924503	1,255503806
P02189	-0,220637484	0,287841701
P04246	-0,445921511	0,344565842
P04178	0,104851607	0,093241918
P02067	-0,099242143	0,086897475
P01846	0,140389968	0,446470315
P01032	-0,095183706	0,184073085
P01025	0,256969697	0,844616215
P00795	-1,09536894	0,707953362
P00690	-0,117324605	0,20710978
P00636	-0,029052819	0,035841958
P01965	-0,162265555	0,166118929
P04366	0,053341407	0,08778164
P04574	0,169854858	0,252069691
P05207	-0,235678037	0,524786911
P12067	-0,069960236	0,129527625
P12026	0,055563431	0,07621234
P11708	-0,01173696	0,015911649
P11493	-0,041299455	0,048302811
P10775	-0,247034543	0,209507364
P10668	-0,245524767	0,336317627
P10173	0,123942346	0,254083649
P63053	-0,263645736	0,581244207
P0C5I2	-0,004586903	0,007232764
P09571	-0,126279156	0,339300332
P08835	-0,123692142	0,491536012
P08132	-0,106119477	0,207366474
P08059	0,00310421	0,004094666
P06867	0,183641506	0,714460464
P06348	-0,101896667	0,177872835
P00571	0,02500126	0,033184163
P12069	-0,432945925	0,620558959
P00503	-0,069690656	0,126391739
P00355	0,054587427	0,059989957
F1SAM7	-0,184814201	0,391537762
F1RWC3	-0,765135691	1,214530619
F1RQM2	-0,041943335	0,067059755

F1RKQ4	0,062957777	0,066202935
D7PF45	0,152627892	0,224716396
D2SW95	0,05537306	0,081473561
O02668	0,143091944	0,467199938
C0HL13	-0,58634161	0,979696514
A7Y521	-0,051691063	0,064868147
A7TX81	0,041504363	0,055341333
A5GFY8	0,231267659	0,203406381
A4Z6H1	-0,145906231	0,2317534
A1YIZ1	-0,346735134	0,322584719
A1XQU1	-0,042130919	0,048067187
B1PK17	-0,012981877	0,007580284
O02705	0,120457045	0,157128051
O02772	0,196636331	0,162406663
O18994	0,029838025	0,043796491
P00348	-0,238504923	0,249349526
P00346	-0,050844996	0,099519787
P00339	-0,41482216	0,369016347
P00336	-0,037160336	0,059481031
P00172	-0,471637184	0,651631809
O97788	-0,138252981	0,253471734
O97763	-0,14167369	0,29383557
O97507	0,111206871	0,240190809
O77591	-0,392974379	0,841124089
O62839	-0,29552503	0,606348592
O46658	-0,413871724	0,628836681
O46560	0,303858396	0,328939065
O46409	0,011542663	0,021840484
O19063	-0,040924909	0,039271098
O19062	0,556775117	0,534998989
P00371	-0,257833442	0,566923626
P79380	0,094806843	0,101136766
P12309	-0,409758041	1,070682675
P14287	-0,469676207	0,516619756
P53603	0,024922284	0,018407522
P52552	-0,333366164	0,562372856
P51779	-0,156071086	0,145415402
P51525	-0,396275655	0,410398084
P50828	-0,02107095	0,060315952
P50584	-0,459974717	0,373006054
P54612	-0,09493543	0,046812271
P50578	0,040558995	0,043871454
P50390	-0,121438422	0,392248694
P50133	0,243016394	0,438303399
P49924	-0,376736624	0,333254272
P49666	0,030256189	0,034813252
P49171	-0,070158808	0,129945567
P48819	0,137845504	0,483338883
P50447	-0,017147558	0,043762315
P59083	0,013254546	0,016352533

P60662	0,027491808	0,036752965
P60982	-0,455422505	0,305078274
P79378	0,181564387	0,487170383
P79376	-0,083746385	0,147497287
Q9TV69	-0,416587178	0,562506982
P79303	-0,309430994	0,289949216
P79273	-0,234810626	0,435281699
P79263	0,380739273	1,086329267
P68137	0,33166921	0,314687054
P63246	-0,468850752	0,684254413
P62936	-0,083447801	0,223135739
P62844	0,320084858	0,617856433
P62831	-0,169032492	0,333259392
P62802	-0,451261292	0,47298989
P62279	0,083612872	0,088500608
P62197	0,248597914	0,436905073
P61220	0,37239131	0,954475349
P47788	-0,11425145	0,168047387
P12682	-0,204291005	0,417885209
P46405	-0,310090999	0,647334255
P43030	-0,254185742	0,576692539
P20305	0,070477343	0,213600616
P19620	0,12521143	0,204648482
P19205	0,60960245	0,60926958
P19133	-0,140582654	0,136699246
P19130	-0,090416084	0,141029547
P18650	-0,183253774	0,242964062
P20735	0,295506722	0,518813391
P18648	0,076154013	0,188861754
P16293	0,214216617	0,419065636
P15468	-0,359505965	0,384664263
P15145	-0,253263901	0,501043157
P14477	1,369161337	0,980727822
P14460	0	NaN
P14332	-0,241892666	0,337909935
P17560	-0,591997187	0,52216151
P22411	-0,270020009	0,403512111
P22412	-0,305777296	0,328990129
P23687	0,677102482	1,27206596
P42831	-0,313432333	0,766535691
P37111	0,080592587	0,094890357
P36968	0,084872595	0,261605759
P32394	0,334125093	0,633880693
P30034	-0,328141593	0,317635064
P29797	-0,26256565	0,570779571
P29700	0,05919615	0,177366807
P29269	0,012376607	0,018987854
P28491	-0,089899855	0,101216159
P27917	-0,032373547	0,044546044
P27485	-0,444460146	0,889645975

P26893	-0,129511402	0,242074823
P26234	0,212477325	0,342371251
P26044	0,377041119	0,342262261
P24853	-0,480310238	1,198422387
P43367	-0,294955379	0,565618117
Q9TVC1	0,082331964	0,06239866

The difference indicates doxycycline vs. control group. Proteins were considered significant if -log10 (p-value) > 1.3. Significant p-values are marked bold.
Abbreviations: N/A = not available
