# Supplementary material for: Doxycycline Alters the Porcine Renal Proteome and Degradome during Hypothermic Machine Perfusion
Source: Curr Issues Mol Biol. 2022 Jan 23;44(2):559–77. doi: 10.3390/cimb44020039 (PMC8928973; doi:10.3390/cimb44020039)
Supplement: Supplementary file 1 [file cimb-44-00039-s001.zip › Table S2.pdf]

Table S2. Identified degradation products pre-reperfusion (T-10).

Accession number	Position in protein	Protein substrate	Difference (DOXY vs Control)	-Log10 (P-value)
P06348	[70-82]	[L].AAAGYDVEKNNSR.[I]	-1,560712537	**2,642160809**
P00348	[149-162]	[T].SSLQITSLANATTR.[Q]	-1,398005133	**1,322692263**
P05024	[214-225]	[N].SSLTGESEPQTR.[S]	-1,577358017	**1,493041642**
Q9TV61	[1377-1386]	[R].TKYETDAIQR.[T]	-1,808785508	**2,079208174**
P80928	[75-87]	[R].SYSKLLCGLLAER.[L]	-1,646971748	**2,727235283**
Q6QAQ1	[107-116]	[T].EAPLNPKANR.[E]	-1,301246082	**1,376620246**
Q2F7Z7	[36-50]	[A].EAFDSVLGDTASCHR.[A]	-1,920492316	**1,97677672**
Q6QAQ1	[234-254]	[S].SSLEKSYELPDGQVITIGNER.[F]	-1,681310138	**2,054281461**
Q2XVP4	[294-308]	[N].ACFEPANQMVKCDPR.[H]	-1,755089222	**2,551668156**
P18648	[71-84]	[L].DNWDSLGSTFTKVR.[E]	-0,7430647	**1,418277225**
P18648	[25-33]	[Q].DDPQSPWDR.[V]	-0,637013122	**1,712241382**
P15145	[895-904]	[S].FSNLIQGVTR.[R]	-0,454979621	**1,518352732**
P04366	[30-43]	[W].FHVAVGSTCPWLKR.[F]	-1,751182336	**3,059128915**
P37111	[7-17]	[R].EGEHPSVTLFR.[Q]	-1,320619237	**2,120694398**
P50447	[25-39]	[A].EGLQGHAVQETDVPR.[H]	-0,598837162	**1,570881288**
P62802	[25-36]	[R].DNIQGITKPAIR.[R]	-1,713573394	**1,87822717**
A5GZW8	[58-70]	[A].SSKAASLHWTGER.[V]	-1,284526976	**3,083745477**
Q6QAQ1	[233-254]	[S].SSSLEKSYELPDGQVITIGNER.[F]	-1,013515505	**3,998571991**
P80021	[254-262]	[R].STVAQLVKR.[L]	-1,913475764	**1,656967207**
P0C5I2	[1-13]	[-].MKALILVGGYGTR.[L]	-1,105147125	**2,314790876**
P04366	[54-62]	[L].MLGEGATER.[E]	-1,610659743	**2,737384354**
Q8MJ14	[7-17]	[R].SAAALAAVAPR.[S]	-1,582820436	**4,082786496**
P20305	[426-441]	[A].QHGMDDDGTGQKQIWR.[I]	-2,206027187	**1,574229013**
Q9GMB0	[26-39]	[A].SPEALPLVNEDVKR.[T]	-2,065934038	**2,481854133**
P50578	[182-203]	[A].VLQVECHPYLAQNELIAHCQAR.[G]	-2,219071925	**1,881590613**
P82460	[2-21]	[M].VKQIESKYAFQEALNSAGEK.[L]	-1,890162164	**1,698424314**
P02067	[2-31]	.VHLSAEEKEAVLGLWGKVNVDEVGGEALG[illegible]	-1,886984597	**1,957582928**
P02067	[2-9]	[M].VHLSAEEK.[E]	-5,766934892	**2,204048969**
P62802	[61-68]	[K].VFLENVIR.[D]	-2,09166705	**3,506601567**
Q95332	[89-108]	[N].YVAEKISGQKVNEAACDIAR.[Q]	-1,715075359	**1,364463643**
Q2XVP4	[357-373]	[N].YQPPTVVPGGDLAKVQR.[A]	-1,654333004	**1,924184518**

P18648	[95-106]	[F].WDNLEKETEALR.[Q]	-1,636753009	**3,587379254**
P80928	[5-12]	[F].VVNTNVPR.[A]	-2,070667709	**1,616961306**
Q2EN81	[28-40]	[L].VRPPVQIYGIEGR.[Y]	-1,00018773	**1,308624724**
Q7M329	[159-173]	[G].EKVQTLGQIELCLTR.[D]	-1,484968709	**2,266661772**
P62279	[4-19]	[R].MHAPGKGLSQSALPYR.[R]	-0,687083237	**1,374127387**
P27917	[24-41]	[A].EDTSLLDKMQDYVKQATR.[T]	-1,425681906	**4,186750763**
A1Z623	[31-39]	[G].AEFSSESCR.[E]	-0,511712297	**2,315988186**
P62936	[9-19]	[F].DIAVDGEPLGR.[V]	-2,276119512	**2,01552347**
P62279	[5-19]	[M].HAPGKGLSQSALPYR.[R]	-2,115887534	**2,640367457**
P01965	[1-11]	[-].VLSAADKANVK.[A]	-6,795758506	**2,364612218**
P08835	[25-33]	[R].DTYKSEIAH.[R]	-0,859031528	**3,278138146**
O02705	[603-613]	[T].STYGWTANMER.[I]	-2,318037288	**1,869960544**
P01965	[1-9]	[-].VLSAADKAN.[V]	-1,893344091	**2,367616504**
P01965	[1-8]	[-].VLSAADKA.[N]	-1,607125107	**1,418865535**
O02668	[68-73]	[Q].STITSR.[M]	-0,68544556	**1,789051215**
P02067	[35-41]	[V].VYPWTQR.[F]	-1,808248777	**1,83524517**
P01965	[1-9]	[-].VLSAADKAN.[V]	-1,358970705	**1,524330774**
P53590	[258-270]	[F].DAKINFDDNAEFR.[Q]	-1,825243677	**1,848371868**
Q2QLE2	[2-21]	[M].GLETEKADVQLFMDDDSYSR.[H]	-1,982213724	**1,872442685**
P26044	[2-8]	[M].PKPINVR.[V]	-1,687644653	**4,202367639**
Q95339	[4-10]	[S].VVPLKDR.[R]	-2,336481591	**2,225997676**
Q66RM2	[1-6]	[-].MNIFDR.[K]	-1,806803008	**2,628088355**
A5GFY8	[450-460]	[N].GAVFRPEVPLR.[R]	-1,805934552	**2,381828361**
P50390	[21-41]	[A].GPAGAGESKCPLMVKVLDAVR.[G]	-2,246241083	**1,32205534**
Q5S3G4	[34-49]	[S].GGGVPTDEEQATGLER.[E]	-0,598022607	**1,603229132**
P00346	[66-74]	[A].ADLSHIETR.[A]	-1,727201056	**2,858683941**
P00503	[2-32]	.APPSVFAEVPQAQPVLVFKLIADFREDPDPF	-2,3434537	**2,22999992**
Q6QAQ1	[29-37]	[R].AVFPSIVGR.[P]	-1,767902708	**3,399500309**
P00889	[28-47]	[S].ASSTNLKDILADLIPKEQAR.[I]	-1,457976823	**2,485301773**
Q95332	[2-8]	[M].APVGDKK.[A]	-1,491582838	**1,473847376**
P51779	[27-36]	[R].ILGGQEAKSH.[E]	-1,871449127	**2,108764982**
Q71LE2	[104-117]	[G].LFEDTNLCAIHAKR.[V]	-1,387377992	**3,150351999**
Q8MJ39	[26-37]	[S].LESYEINPFLNR.[R]	-1,625360524	**1,740604189**

P06348	[69-82]	[A].LAAAGYDVEKNNSR.[I]	-1,484843774	**3,673825235**
Q0MVN8	[46-55]	[C].GVNPVDTYIR.[S]	-1,807200983	**2,844656211**
P28491	[21-36]	[T].IYFKEQFLDGDGWTDR.[W]	-1,909791018	**1,495952955**
Q2EN81	[27-40]	[K].LVRPPVQIYGIEGR.[Y]	-0,555152563	**1,402952121**
P02554	[65-77]	[I].LVDLEPGTMDSVR.[S]	-1,469864128	**2,593175747**
Q9N0F1	[196-222]	P].LTSKPVSAVKPTAAPPVAEPGAVKGLR.[A	-1,31833589	**3,239650676**
O19069	[143-154]	[C].ITEGIPQQDMVR.[V]	-1,826735292	**1,776059263**
P00346	[25-52]	N].AKVAVLGASGGIGQPLSLLLKNSPLVSR.[L	-1,490727906	**2,889418975**
Q29594	[2-13]	[M].PFSNSHNTLKLR.[F]	-1,694641679	**3,849926512**
Q71LE2	[19-27]	[R].KQLATKAAR.[K]	-2,001451055	**1,70078633**
P42174	[1-15]	[-].SEAAADREDDPNFFK.[M]	-0,819222415	**2,184775123**
Q29548	[25-44]	[T].SGAESLGLWPLPFAVDISPR.[S]	-4,343631479	**1,854076581**
P08059	[107-124]	[R].SNTPILVDGKDVMPEVNR.[V]	-2,052831624	**2,754362612**
P80928	[2-12]	[M].PMFVVNTNVPR.[A]	-1,335861833	**2,580905544**
A5GZW8	[59-70]	[S].SKAASLHWTGER.[V]	-1,370989637	**2,443379217**
Q0QF01	[199-207]	[H].SLLHTLYGR.[S]	-1,899500207	**1,706593802**
Q9MZ15	[278-294]	[K].SINAGGHKLGLALELEA.[-]	-2,088228452	**2,307979309**
P05024	[226-238]	[R].SPDFTNENPLETR.[N]	-1,420108311	**2,702770471**
P02554	[138-156]	[H].SLGGGTGSGMGTLLISKIR.[E]	-1,462349517	**2,740221126**
P16276	[35-44]	[M].SHFEPHEYIR.[Y]	-0,434934418	**1,728515867**
P50441	[400-413]	[N].SLGGGFHCWTCDVR.[R]	-2,048751049	**1,786179893**
P01025	[747-762]	[R].SDLDEEIIPEEDIISR.[S]	-2,295719484	**1,378300414**
Q0QF01	[346-352]	[R].SMTLEIR.[E]	1,698009957	**1,314408669**
Q6PQZ1	[237-243]	[R].SSDLTDR.[V]	1,310458711	**1,354905389**
P29700	[291-304]	[P].MVVAVPPGIPPVHR.[S]	1,402346692	**1,430173616**
Q5S1U1	[29-38]	[R].LFDQAFGLPR.[L]	1,700905673	**1,488847957**
Q6QAQ1	[89-95]	[H].TFYNELR.[V]	1,923208321	**1,997840646**
P80021	[47-58]	[T].GTAEVSSILEER.[I]	1,428428321	**1,598018619**
C0HL13	[4632-4647]	[A].YSATEDTFKDTANLVR.[E]	1,547439336	**1,326061949**
Q29307	[25-34]	[G].FSSDTPEGVR.[S]	1,567004002	**1,510279772**
C0HL13	[26-34]	[G].RECLGNEFR.[C]	1,436112459	**1,368614695**
Q6QAQ1	[8-28]	[A].LVVDNGSGMCKAGFAGDDAPR.[A]	1,876470239	**1,500093482**
P81045	[1-22]	[-].PGLAAAIPAPPESQEKKPLKPC.[C]	1,624786617	**1,522473232**

Q0QF01	[45-75]	AKVSDAISTQYPVVDHEFDAVVVGAGGAGLI	1,717638768	**1,884676373**
P04366	[18-24]	[Q].ENFDLSR.[I]	1,810350493	**1,358185875**
P13618	[1-9]	[-].NKELDPVQK.[L]	1,524131012	**1,47243237**
P06348	[68-82]	[K].ALAAAGYDVEKNNSR.[I]	1,439280286	**1,467243342**
O97763	[20-25]	[A].EPVHFR.[D]	1,543358988	**1,38542213**
P80928	[3-12]	[P].MFVVNTNVPR.[A]	1,615357551	**1,521677122**
P17741	[2-10]	[M].GKGDPNKPR.[G]	1,662370608	**1,594144723**
P83662	[1-8]	[-].MKIDIHSH.[I]	1,525994961	**1,616722132**
D0VWV4	[30-40]	[P].LGTTAKEEMER.[F]	0,052958768	**0,091218558**
P79273	[25-38]	[R].LHTIFQSVELPETY.[Q]	0	NaN
P79273	[25-42]	[R].LHTIFQSVELPETYQMLR.[Q]	0	NaN
P79273	[25-42]	[R].LHTIFQSVELPETYQMLR.[Q]	0	NaN
P79273	[25-42]	[R].LHTIFQSVELPETYQMLR.[Q]	0	NaN
Q75NG9	[87-95]	[A].LIDSHFEAR.[K]	0	NaN
Q6QAQ1	[231-254]	[A].ASSSSLEKSYELPDGQVITIGNER.[F]	0,557351068	0,19269097
P51779	[27-38]	[R].ILGGQEAKSHER.[P]	-0,014566591	0,015488953
Q5S3G4	[32-49]	[M].ASGGGVPTDEEQATGLER.[E]	-0,246069202	0,614934144
P51779	[27-40]	[R].ILGGQEAKSHERPY.[M]	1,185698828	0,693198842
Q6Q7J2	[319-328]	[Q].IIIPQNQVNR.[K]	-0,527571748	0,15609141
Q4FAT7	[23-35]	[A].LLQGGMLYPQESR.[S]	1,045750506	0,487498147
P80021	[135-149]	[T].GAIVDVPVGEELLGR.[V]	0,165239843	0,290644914
P10173	[1-7]	[-].ASQDSFR.[I]	-0,343005993	1,125310557
Q8WN93	[23-39]	[A].ETEEENPDDLIQLTVTR.[N]	0	NaN
Q4KRV1	[27-39]	[A].ASQIEDQAEQFFR.[S]	1,337281925	1,261205516
A5GZW8	[57-70]	[Q].ASSKAASLHWTGER.[V]	-0,104704172	0,165506811
P00889	[26-47]	[H].ASASSTNLKDILADLIPKEQAR.[I]	0,042087445	0,038031939
Q8WNV7	[258-278]	[C].SEDASYITGETVVVGGGTASR.[L]	0	NaN
Q9MYY8	[3-33]	.GAEELPEMYDYDLIIIGGGSGGLAAAKEAAR	0	NaN
Q29308	[2-16]	[M].PGVTVKDVNQQEFVR.[A]	-0,14947135	0,332989415
Q07717	[83-100]	[Y].LLVHTEFTPNAVDQYSCR.[V]	0	NaN
P50828	[254-271]	[D].LVLTALLSDNHGATYAFR.[G]	1,107670577	0,484477432
C0HL13	[345-365]	[R].SCVDFNDCQIWGICDHFCEDR.[I]	0	NaN
Q6QAQ1	[8-28]	[A].LVVDNGSGMCKAGFAGDDAPR.[A]	0,057906497	0,076236438

Q6QAQ1	[8-28]	[A].LVVDNGSGMCKAGFAGDDAPR.[A]	0,153625904	0,217565304
P29700	[286-304]	[S].LVVGPMVVAVPPGIPPVHR.[S]	0,91563229	0,575106459
Q29318	[11-20]	[S].LVVHGPGDLR.[L]	1,608525743	0,754411814
P02067	[15-31]	[G].LWGKVNVDEVGGEALGR.[L]	-0,523630758	1,217532812
P50441	[218-235]	[T].MADELYDQDYPIYSVEDR.[H]	0	NaN
P53590	[39-47]	[W].LNLQEYQSK.[K]	0,013340628	0,020101512
P02067	[32-41]	[R].LLVVYPWTQR.[F]	-0,19225679	0,252525747
Q7M329	[185-200]	[G].LEICEDGPVFYPPPKE.[-]	0	NaN
P02067	[84-105]	[K].GTFAKLSELHCDQLHVDPENFR.[L]	0	NaN
P63053	[57-72]	[L].SDYNIQKESTLHLVLR.[L]	-0,275985539	0,1804139
Q08094	[211-227]	[M].GTNKCASQVGMTAPGTR.[R]	0,888815373	0,527307146
D0VWV4	[31-40]	[L].GTTAKEEMER.[F]	-0,103667976	0,236371968
Q9N0F1	[122-145]	[N].GVIEALLVPDGGKVEGGTPLFTLR.[K]	0	NaN
Q1W0Y2	[17-25]	[R].SHYEEGPGK.[N]	1,702573894	1,090329627
P33198	[142-157]	[R].HAHGDQYKATDFVVDR.[A]	0,234163067	0,129284767
P29700	[18-25]	[P].HGPILGYR.[E]	-1,395263314	0,899045994
P79263	[28-46]	[A].HKNDINIYSLTVDSKVSSR.[F]	-0,498972431	0,444424954
P50828	[24-39]	[A].HPLSLTAGPKHGAEGR.[N]	-2,133763423	1,199346001
P41367	[24-53]	.HTKAVPQCEPGSGFSFELTEQQKEFQATAR	0,041761861	0,035042591
Q0MVN8	[193-201]	[N].GAHEVFNHR.[E]	0	NaN
F1RKQ4	[40-48]	[R].SDLDSLKGR.[V]	0,19727414	0,427013784
P27917	[21-41]	[A].IEAEDTSLLDKMQDYVKQATR.[T]	-0,430011646	0,569493173
P80229	[264-275]	[N].LELLEVNVHLPR.[F]	-0,773129957	0,356350541
Q08092	[209-226]	[M].GTNKGASQAGMTAPGTKR.[Q]	2,003579909	0,971096588
Q71LE2	[96-117]	[E].ASEAYLVGLFEDTNLCAIHAKR.[V]	0	NaN
Q29268	[2-20]	[M].APSGLKAVVGEKILSGVIR.[S]	0,147498642	0,087388746
P00503	[2-26]	[M].APPSVFAEVPQAQPVLVFKLIADFR.[E]	-0,464565904	0,85674383
P80021	[62-73]	[G].ADTSVDLEETGR.[V]	-0,016549936	0,027914547
P79382	[2-9]	[M].ADLTELMK.[N]	-0,293077851	0,113233373
P50441	[52-82]	ADDKATDPLPKDCPVSSYNEWDPLEEVIVGF	0	NaN
Q6QAQ1	[295-312]	[Y].ANTVLSGGTTMYPGIADR.[M]	0	NaN
P01965	[12-31]	[K].AAWGKVGGQAGAHGAEALER.[M]	0,225737274	0,069567992
P01965	[12-31]	[K].AAWGKVGGQAGAHGAEALER.[M]	0	NaN

P00371	[84-99]	[N].AANMGLTPVSGYNLFR.[E]	0	NaN
P06348	[71-82]	[A].AAGYDVEKNNSR.[I]	-0,316229577	0,596613154
P50441	[51-82]	.ADDKATDPLPKDCPVSSYNEWDPLEEVIVG	0,333055536	0,353039935
P80147	[31-54]	[Q].AAAKVDVEFDYDGPLMKTEVPGPR.[S]	-0,026993918	0,025257682
Q8SPS7	[19-49]	AETGNEATDATDDSCPKPPEIPKGYVEHMVI	0	NaN
Q8SPS7	[19-49]	AETGNEATDATDDSCPKPPEIPKGYVEHMVI	-1,949089944	0,842753274
Q8SPS7	[19-49]	AETGNEATDATDDSCPKPPEIPKGYVEHMVI	0	NaN
P14460	[1-17]	[-].AEVQDKGEFLAEGGGVR.[-]	0,277521491	0,098846007
Q8MK48	[2-15]	[M].AFNDLLLQVGGVGR.[F]	-0,195117365	0,269631101
P50390	[23-41]	[P].AGAGESKCPLMVKVLDAVR.[G]	0,848734334	0,727277385
P47788	[8-25]	[C].AGDALDVAAPCSAVNYLR.[W]	0	NaN
Q04967	[225-238]	[T].AGDTHLGGEDFDNR.[L]	1,233244999	0,870102007
Q6QAQ1	[19-28]	[K].AGFAGDDAPR.[A]	0,025108458	0,027364334
Q06AU5	[2-24]	[M].AGGGAGDPGQGAAAAAAAAPETR.[E]	0	NaN
Q2EN81	[25-40]	[F].AKLVRPPVQIYGIEGR.[Y]	0,290101701	0,239436237
Q1W0Y2	[17-36]	[R].SHYEEGPGKNLPFSVENKWR.[L]	0,14025234	0,156563063
Q0QF01	[45-75]	AKVSDAISTQYPVVDHEFDAVVVGAGGAGLI	0	NaN
P14477	[1-13]	[-].AIDYDEDEDGRPK.[V]	-0,993182519	0,615062858
P14477	[1-19]	[-].AIDYDEDEDGRPKVHVDAR.[-]	-1,07978946	0,610983481
C0HL13	[3938-3966]	.CISQHLVCDDVDDCGDHFDETGCNTGEER	0	NaN
P63221	[4-15]	[N].DAGEFVDLYVPR.[K]	-1,048801403	1,032267166
P02067	[6-31]	S].AEEKEAVLGLWGKVNVDEVGGEALGR.[L	0,763056417	0,2886696
P12037	[51-66]	[L].ARFAQEAEAARVELQK.[K]	-0,253664655	1,029870899
Q1W0Y2	[17-29]	[R].SHYEEGPGKNLPF.[S]	-0,159911015	0,251558473
Q8SPS7	[19-41]	[A].AETGNEATDATDDSCPKPPEIPK.[G]	-1,594098469	1,288334453
Q007T0	[29-38]	[G].AQTAAATAPR.[I]	-0,45006098	0,168226261
P80021	[447-463]	[F].AQFGSDLDAATQQLLSR.[G]	0,352447994	0,120602551
Q28943	[2-21]	[M].APVLSKDVADIESILALNPR.[T]	-0,340441884	0,738858437
Q95283	[30-41]	[K].SEDYALPVYVDR.[R]	-0,438531436	1,125927577
Q0MVN8	[188-201]	[N].IVLQNGAHEVFNHR.[E]	-1,222350786	0,447174499
Q29371	[43-53]	[C].APPTAYIDFAR.[Q]	-0,39276882	0,189316359
Q9TV69	[27-37]	[R].SEHQVVAVAAR.[D]	-0,137689206	0,222290549
Q95339	[2-10]	[M].ASVVPLKDR.[R]	0,003668867	0,00548501

P50441	[42-62]	[A].ATASSGNSCAADDKATDPLPK.[D]	0	NaN
A1XQT2	[2-12]	[M].ATSSLTKPQMR.[W]	0,011056572	0,012681101
Q29550	[15-33]	[S].ATWAGQPASPPVVDTAQGR.[V]	0	NaN
Q4U116	[1039-1066]	Y].SEKVPSIKIPMDIMEQQPFLSDSKPSDR.[E	1,584090573	0,89086262
P29700	[294-304]	[V].AVPPGIPPVHR.[S]	0	NaN
P01965	[13-31]	[A].AWGKVGGQAGAHGAEALER.[M]	0,109740973	0,045009399
Q19KI0	[2-8]	[M].AWNTNLR.[W]	1,40756988	1,230675058
P23695	[25-48]	[A].AYRPSETLCGGELVDTLQFVCGDR.[G]	0	NaN
C0HL13	[2757-2773]	[R].CDHYNDCGDNSDESGCR.[F]	0	NaN
P08835	[86-105]	[N].CDKSIHTLFGDKLCAIPSLR.[E]	0	NaN
C0HL13	[3951-3966]	[D].CGDHFDETGCNTGEER.[S]	0	NaN
Q6QAQ1	[17-28]	[M].CKAGFAGDDAPR.[A]	0	NaN
Q6QAQ1	[17-28]	[M].CKAGFAGDDAPR.[A]	0,12236416	0,147030881
C0HL13	[3645-3675]	IPQSWKCDVDNDCGDYSDEPLQECMGPAY	0	NaN
P81405	[7-19]	[D].CIQMVTDLQNAVR.[T]	0,414687121	0,199922294
Q04967	[434-449]	[Y].SDNQPGVLIQVYEGER.[A]	1,383903056	0,784131171
O62839	[411-424]	[S].APEQTHSALEHCTR.[Y]	0	NaN
O02705	[599-613]	[C].CIVTSTYGWTANMER.[I]	0	NaN
P80021	[276-301]	V].SATASDAAPLQYLAPYSGCSMGEYFR.[D	0	NaN
Q8SPS7	[19-41]	[A].AETGNEATDATDDSCPKPPEIPK.[G]	0	NaN
Q71LE2	[101-117]	[Y].LVGLFEDTNLCAIHAKR.[V]	0,964629949	0,408106761
P50828	[28-39]	[S].LTAGPKHGAEGR.[N]	-1,19972081	0,751419764
Q29092	[647-660]	[A].LVASQYGWSGNMER.[I]	-0,007775045	0,012091727
C0HL13	[3193-3203]	[T].NIEPYLIFSNR.[Y]	0,97403881	0,571322182
Q95333	[22-29]	[G].HPKSLNQR.[E]	0	NaN
Q1KYT0	[247-253]	[V].AASEFYR.[N]	-0,071248551	0,032138324
P26042	[481-495]	[Q].DEQDENGAEASADLR.[A]	0	NaN
Q29048	[72-81]	[S].GVSVGDPVLR.[T]	0,99336188	0,378699702
P00571	[116-128]	[L].LYVDAGPETMTKR.[L]	1,118320274	0,585980499
P09571	[455-462]	[K].SCHTAVDR.[T]	0	NaN
Q28955	[2-8]	[M].PLLVEGR.[R]	-0,547004613	0,241874577
Q19KI0	[2-8]	[M].AWNTNLR.[W]	-0,100607813	0,093780421
A1XQT2	[4-12]	[T].SSLTKPQMR.[W]	1,515770865	0,673031273

O02705	[675-688]	[S].SGFSLEDPQTHANR.[I]	0	NaN
P16225	[138-143]	[R].FLLSKR.[G]	1,607282619	1,000631483
Q1KYT0	[373-400]	R].SGETEDTFIADLVVGLCTGQIKTGAPCR.[S	-0,680436272	0,170972823
P50828	[30-39]	[T].AGPKHGAEGR.[N]	-0,047499102	0,026260501
Q6RVA9	[3-19]	[S].GGKYVDSEGHLYTVPIR.[E]	0,159079353	0,10037102
C0HL13	[4478-4499]	[R].SGEDVNMDIGVSGFGPESAIDR.[S]	0	NaN
P01025	[958-997]	ADLSDQVPDTESETKILLQGTPVAQMVEDA	0	NaN
P08835	[27-34]	[T].YKSEIAHR.[F]	-0,122234205	0,035092897
P80021	[285-301]	[P].LQYLAPYSGCSMGEYFR.[D]	0	NaN
P80021	[292-301]	[Y].SGCSMGEYFR.[D]	-0,192950417	0,248544746
P62802	[81-93]	[K].TVTAMDVVYALKR.[Q]	-0,781333157	0,378008133
P00371	[93-99]	[V].SGYNLFR.[E]	0,140064605	0,11361275
P29700	[305-310]	[R].SHYDLR.[H]	-0,361398717	0,170513735
Q6SEG5	[1-19]	[-].MQLKPMEINPEMLNKVLTR.[L]	0	NaN
Q3ZD69	[12-25]	[R].SGAQASSTPLSPTR.[I]	0	NaN
P50441	[39-55]	[S].TQAATASSGNSCAADDK.[A]	0	NaN
P01025	[671-677]	[S].VQLMEKR.[M]	-0,921331336	0,512793523
Q71LE2	[58-64]	[K].STELLIR.[K]	-0,327249855	0,816171061
P80021	[448-463]	[A].QFGSDLDAATQQLLSR.[G]	1,020762942	0,382050838
P50441	[38-62]	[Q].STQAATASSGNSCAADDKATDPLPK.[D]	0	NaN
Q5S3G4	[33-49]	[A].SGGGVPTDEEQATGLER.[E]	-0,348983729	1,17632912
Q6QAQ1	[300-312]	[LM].SGGTTMYPGIADR.[M]	0,005567441	0,005364073
P80041	[336-347]	[H].SHQGSGLITDYR.[H]	-0,33767869	0,614878964
Q9TSX9	[3-22]	[P].GGLLLGDEAPNFEANTTIGR.[I]	0	NaN
Q6QAQ1	[301-312]	[S].GGTTMYPGIADR.[M]	1,38283866	0,72838642
A1E295	[78-87]	[D].MILPKSFDAR.[E]	1,26389769	0,619042145
P00791	[16-23]	[C].LVKVPLVR.[K]	0,562471334	0,145348466
P08835	[310-318]	[K].SHCIAEAKR.[D]	-1,931061736	1,285952033
P00636	[144-158]	[N].STDEPSEKDALQPGR.[N]	1,181412056	0,538401368
Q7YS91	[40-60]	[L].TSSATSPSSHLPGSLSELVER.[G]	0	NaN
P26042	[144-156]	[K].SGYLAGDKLLPQR.[V]	-0,175749813	0,286522524
Q29308	[3-16]	[P].GVTVKDVNQQEFVR.[A]	0,88777541	0,49668633
Q71LE2	[107-117]	[E].DTNLCAIHAKR.[V]	0,726974985	0,336307348

P08059	[22-27]	[R].SDLNLR.[R]	-0,156587642	0,34281206
P80928	[64-74]	[H].SIGKIGGAQNR.[S]	-0,061101141	0,068196973
P81140	[12-20]	[K].SSRPEFDWR.[D]	0,680788001	0,341911796
P00795	[1-8]	[-].GPIPEVLK.[N]	0,18104517	0,124910443
Q9GJT2	[65-86]	[K].SGYHQAASEHGLVVIAPDTSPR.[G]	0	NaN
P62802	[57-68]	[R].GVLKVFLENVIR.[D]	1,113161556	0,474032225
P01025	[967-997]	⊃QVPDTESETKILLQGTPVAQMVEDAIDGDI	0	NaN
Q29550	[17-33]	[T].WAGQPASPPVVDTAQGR.[V]	0	NaN
Q07717	[21-32]	[A].VARPPKVQVYSR.[H]	-0,60770659	0,273675286
P01965	[1-7]	[-].VLSAADK.[A]	-0,116435428	0,079138049
C0HL13	[4036-4044]	[R].SMSEHYGER.[C]	0	NaN
Q4FAT7	[23-35]	[A].LLQGGMLYPQESR.[S]	-0,472638006	0,2178124
C0HL13	[3068-3078]	[K].EETTCPPHQFR.[C]	1,05929852	0,552757079
Q8WNV7	[21-27]	[M].ASTGVER.[R]	0,813716566	0,432517787
O19069	[275-295]	[N].SGPKSKPVVSFIAGLTAPPGR.[R]	0,138684837	0,071284543
P80021	[195-204]	[K].AVDSLVPIGR.[G]	-0,060786208	0,038034622
Q04967	[68-74]	[N].TVFDAKR.[L]	-0,51390031	0,147733448
Q04967	[39-51]	[R].TTPSYVAFTDTER.[L]	0	NaN
Q8WN98	[1-20]	[-].MKDPDPSQVYRPDMDPEAAK.[D]	0,193278606	0,083216654
P09571	[116-123]	[RK].SCHTGLGR.[S]	-0,670831223	0,207198503
P11708	[143-157]	[K].SAPSIPKENFSCLTR.[L]	0,460838958	0,315400801
Q6QAQ1	[178-183]	[R].LDLAGR.[D]	0,876339202	0,405587454
C0HL13	[4633-4647]	[Y].SATEDTFKDTANLVR.[E]	0,463626941	0,240608672
		[C].GLLAQR.[R]	0	NaN
C0HL13	[1360-1382]	[N].GGCTHLCIQGPFGAQCECPLGYR.[L]	0	NaN
P41367	[380-392]	[N].GFNTEYPVEKLMR.[D]	-1,574207157	0,585545137
Q9TSX9	[4-22]	[G].GLLLGDEAPNFEANTTIGR.[I]	0	NaN
Q52NJ4	[2-8]	[M].GLLSILR.[K]	-0,168792302	0,560030529
O46427	[293-317]	N].GIPYWIVKNSWGPQWGMNGYFLIER.[G	0	NaN
P81140	[363-372]	[N].GISDEYHVIR.[H]	-1,351705473	0,802617096
Q4U116	[417-438]	[N].GDTPHDGGHGGGGHADCEELQR.[T]	0	NaN
Q6QAQ1	[53-62]	[S].YVGDEAQSKR.[G]	-0,568609172	0,233300763
Q6QAQ1	[13-28]	[N].GSGMCKAGFAGDDAPR.[A]	-1,119002412	0,313828406

P04366	[8-24]	[V].LTLPNDIQVQENFDLSR.[I]	-1,672005719	1,281672581
P53590	[39-56]	[W].LNLQEYQSKKLMSDNGVK.[V]	1,317312102	0,819788203
Q95334	[603-618]	[N].GNAFLKINPDHIGFYR.[V]	-0,297622796	0,577346489
P80021	[408-416]	[A].INVGLSVSR.[V]	-0,074659863	0,030827912
Q0MVN8	[24-43]	[K].LQSDVAIPIPKDNQVLIKVH.[A]	0	NaN
P02067	[4-31]	].LSAEEKEAVLGLWGKVNVDEVGGEALGR.	0,315963487	0,128918764
P02067	[89-105]	[K].LSELHCDQLHVDPENFR.[L]	-0,714123541	0,375477874
P50828	[26-39]	[P].LSLTAGPKHGAEGR.[N]	-0,352558265	0,312904184
Q29318	[9-20]	[N].LSLVVHGPGDLR.[L]	0,477063701	0,295533262
P80147	[28-54]	[illegible]].ISQAAAKVDVEFDYDGPLMKTEVPGPR.[[illegible]	0,281065786	0,486315214
Q2XVP4	[286-308]	[Q].LSVAEITNACFEPANQMVKCDPR.[H]	0	NaN
P80021	[61-73]	[L].GADTSVDLEETGR.[V]	0	NaN
Q6QAQ1	[105-116]	[L].LTEAPLNPKANR.[E]	0,979752081	0,401263557
P50441	[49-82]	[illegible]AADDKATDPLPKDCPVSSYNEWDPLEEVIV[illegible]	-0,517550215	0,190302496
Q8WNQ7	[377-386]	[R].LTERPIFYYR.[G]	-0,765031833	0,401511087
Q29195	[79-88]	[K].SCGKDGFHIR.[V]	0,939218998	0,437079888
Q8SPS7	[103-120]	[R].IMGGSLDAKGSFPWQAKM.[I]	-0,375184755	0,178114592
P04366	[10-24]	[T].LPNDIQVQENFDLSR.[I]	1,059107073	0,619203747
P80928	[82-87]	[C].GLLAER.[L]	-0,591770291	1,175738034
P81405	[1-9]	[-].GDVCQDCIQ.[M]	0	NaN
Q6QAQ1	[273-290]	[C].GIHETTFNSIMKCDVDIR.[K]	-0,459817442	0,502502427
O62839	[306-320]	[H].SEYPLIPVGKLVLNR.[N]	0,109858135	0,107174132
Q6QAQ1	[273-290]	[C].GIHETTFNSIMKCDVDIR.[K]	-0,954700155	0,490014089
Q4U116	[336-342]	[K].SYHEIGR.[A]	-0,105466454	0,190788113
P14460	[9-17]	[E].FLAEGGGVR.[-]	-0,433404783	0,140016993
P29797	[2-13]	[M].GCLGNSKTEDQR.[N]	0,706715333	0,272042084
P01965	[15-31]	[W].GKVGGQAGAHGAEALER.[M]	0	NaN
P05027	[176-182]	[C].VIIKLNR.[V]	0,389803936	0,288754312
P50390	[26-41]	[A].GESKCPLMVKVLDAVR.[G]	-0,770071208	0,777642075
O11780	[24-38]	[A].GPAKSPYQLVLQHSR.[L]	0	NaN
Q29550	[19-33]	[A].GQPASPPVVDTAQGR.[V]	-0,769057848	0,392123675
P81405	[1-19]	[-].GDVCQDCIQMVTDLQNAVR.[T]	-0,203713166	0,359521641
P29700	[335-356]	[K].GAQPSIPAADGSVPVVRPCPGR.[I]	0	NaN

P05024	[208-225]	[HN].GCKVDNSSLTGESEPQTR.[S]	-1,023278306	0,307402915
P81649	[91-118]	].GQNNCHQSAKPVSLTQCSFTGGNYPNCR.	0	NaN
Q2XVP4	[54-64]	[F].SETGAGKHVPR.[A]	0,972939241	0,573642658
P01965	[19-31]	[G].GQAGAHGAEALER.[M]	-0,15368211	0,20687261
P81405	[1-16]	[-].GDVCQDCIQMVTDLQN.[A]	0	NaN
P81405	[1-19]	[-].GDVCQDCIQMVTDLQNAVR.[T]	1,051883368	0,591952251
P81405	[1-19]	[-].GDVCQDCIQMVTDLQNAVR.[T]	-0,18785601	0,365061479
O46427	[114-123]	[T].GPYPPSMDWR.[K]	-0,082386608	0,14418934
Q6QAQ1	[20-28]	[A].GFAGDDAPR.[A]	0,094161535	0,052063221
P08835	[29-34]	[K].SEIAHR.[F]	-1,294072449	0,802243959
P80021	[192-204]	[T].GIKAVDSLVPIGR.[G]	-0,035530999	0,033882154
Q5S3G4	[35-49]	[G].GGVPTDEEQATGLER.[E]	1,245392568	0,928271235
A1XQU5	[2-17]	[M].GKFMKPGKVVLVLAGR.[Y]	-0,239908955	0,521642624
P36968	[74-89]	[U].GKTEVNYTQLVDLHAR.[Y]	0	NaN
Q06AU5	[7-24]	[A].GDPGQGAAAAAAAAPETR.[E]	1,370172222	0,686459676
P01965	[15-31]	[W].GKVGGQAGAHGAEALER.[M]	-0,038464049	0,01393965
Q71LE2	[103-117]	[V].GLFEDTNLCAIHAKR.[V]	0	NaN
Q5U9S1	[109-114]	[N].LEGQIR.[I]	0	NaN
P80229	[265-275]	[L].ELLEVNVHLPR.[F]	1,454237059	0,756551692
Q71LE2	[74-84]	[R].EIAQDFKTDLR.[F]	0,086917515	0,050120277
P28491	[18-36]	[A].EPTIYFKEQFLDGDGWTDR.[W]	-0,032737242	0,043284872
Q28943	[4-21]	[P].VLSKDVADIESILALNPR.[T]	-0,128641885	0,199774378
P79384	[30-38]	[C].TQPVSVNER.[I]	-0,405147133	0,839217283
P02543	[5-12]	[R].TVSSSSYR.[R]	1,036259316	0,491062982
A5A8V7	[22-38]	[G].VFQHGKVEIIANDQGNR.[T]	1,068022077	0,606220232
P05024	[211-225]	[K].VDNSSLTGESEPQTR.[S]	0	NaN
P00346	[27-52]	[K].VAVLGASGGIGQPLSLLLKNSPLVSR.[L]	-1,071959782	0,539063801
P08835	[26-34]	[D].TYKSEIAHR.[F]	-0,60776943	0,440535657
P01965	[41-50]	[K].TYFPHFNLSH.[G]	0	NaN
Q6QAQ1	[297-312]	[N].TVLSGGTTMYPGIADR.[M]	0	NaN
Q6Q2J0	[269-291]	[Y].VISAIPPTLTAKIHFRPELPSER.[N]	0	NaN
Q6QAQ1	[297-312]	[N].TVLSGGTTMYPGIADR.[M]	-1,061498712	0,332479352
Q29554	[111-125]	[C].TTSQEVTQISQEAQR.[T]	0	NaN

A1XQU1	[44-62]	[G].TTIAGVVYKDGIVLGADTR.[A]	0,005016889	0,010413099
P62936	[2-19]	[M].VNPTVFFDIAVDGEPLGR.[V]	-0,039512375	0,07051896
Q6QAQ1	[148-173]	[R].TTGIVMDSGDGVTHTVPIYEGYALPH.[A	0	NaN
P00348	[148-162]	[N].TSSLQITSLANATTR.[Q]	-1,457982769	0,709425834
P01965	[17-31]	[K].VGGQAGAHGAEALER.[M]	0,982093333	0,501785042
A1XQR6	[5-18]	[A].VGPYGQSQPSCFDR.[V]	1,429697827	0,66344165
P00355	[4-11]	[K].VGVNGFGR.[I]	-0,276675324	0,578607755
Q9N1F5	[8-25]	[R].SLGKGSAPPGPVPEGLIR.[V]	-0,180954568	0,097542643
O02772	[23-31]	[K].SIGVGFATR.[Q]	-0,037682836	0,074262347
P02067	[2-31]	.VHLSAEEKEAVLGLWGKVNVDEVGGEALGI	0,070948462	0,02754479
Q07717	[85-100]	[L].VHTEFTPNAVDQYSCR.[V]	0	NaN
Q06AT9	[2-11]	[M].VKLFIGNLPR.[E]	-0,254844934	0,592353008
C0HL13	[2398-2430]	SLHFDPEDYNVPFTAISVEETAVAVDYDSIDN	0	NaN
P82460	[2-21]	[M].VKQIESKYAFQEALNSAGEK.[L]	0,083995541	0,039213767
P00355	[2-11]	[M].VKVGVNGFGR.[I]	0	NaN
P00355	[2-11]	[M].VKVGVNGFGR.[I]	0,210816347	0,453183166
P02543	[54-64]	[Y].TSSPGGVYATR.[S]	-0,169763804	0,239449237
P00355	[2-11]	[M].VKVGVNGFGR.[I]	0,136790529	0,236890594
Q3ZD69	[51-60]	[R].SLETENAGLR.[L]	-0,414269466	0,21015826
Q4U116	[407-438]	GGENVQMNGDTPHDGGHGGGGHADCEEI	0	NaN
P02543	[87-100]	[F].SLADAINTEFKNTR.[T]	-1,765802957	0,582186908
P29700	[287-304]	[L].VVGPMVVAVPPGIPPVHR.[S]	0,360148941	0,233891748
P20305	[43-62]	[M].VVEHPEFLKAGKEPGLQIWR.[V]	0,086838146	0,05802544
Q6QAQ1	[9-28]	[L].VVDNGSGMCKAGFAGDDAPR.[A]	0,834113285	1,096683691
P29700	[292-304]	[M].VVAVPPGIPPVHR.[S]	0,184396657	0,296655955
P02067	[134-147]	[K].VVAGVANALAHKYH.[-]	0	NaN
Q0QF01	[47-75]	.VSDAISTQYPVVDHEFDAVVVGAGGAGLR.	0	NaN
P00377	[1-28]	.VRPLNCIVAVSQNMGIGKNGDLPWPPLR.[	1,155889331	0,610620363
P04366	[7-24]	[P].VLTLPNDIQVQENFDLSR.[I]	0,702342272	0,374722511
Q4U116	[413-438]	].VQMNGDTPHDGGHGGGGHADCEELQR.	0	NaN
Q4U116	[413-438]	].VQMNGDTPHDGGHGGGGHADCEELQR.	0	NaN
P04366	[16-24]	[Q].VQENFDLSR.[I]	-0,689684982	0,422575796
Q9MYT8	[2-15]	[M].VPPVQVSPLIKLGR.[Y]	-0,112690405	0,308759744

P80031	[3-11]	[P].YTITYFPVR.[G]	-0,554694706	0,277657243
P29700	[16-25]	[A].VPHGPILGYR.[E]	-0,315160145	1,219636677
Q29318	[12-20]	[L].VVHGPGDLR.[L]	0	NaN
B1PK17	[169-188]	[R].SIAKYPVGIEVGPQPQGVLR.[A]	0,979884863	0,508056989
P02554	[101-121]	[N].WAKGHYTEGAELVDSVLDVVR.[K]	0,884699943	0,58275179
P67937	[6-12]	[N].SLEAVKR.[K]	0,068239504	0,080774514
P01965	[14-31]	[A].WGKVGGQAGAHGAEALER.[M]	0,116246124	0,053530772
P18648	[22-33]	[F].WQQDDPQSPWDR.[V]	-0,667886954	1,116638532
C0HL13	[85-102]	[Y].WVCDGEEDCSNGADEHQR.[C]	0	NaN
P06348	[74-82]	[G].YDVEKNNSR.[I]	-0,011480117	0,016775217
Q6QAQ1	[240-254]	[S].YELPDGQVITIGNER.[F]	1,116697709	0,440065429
C0HL13	[4551-4584]	SPVNPDELAPDTKPASPSADETQVTKWNIF	0	NaN
Q29545	[261-273]	[C].YLAQVPSHAVVAR.[S]	1,230435185	0,643253796
Q1W0Y2	[17-34]	[R].SHYEEGPGKNLPFSVENK.[W]	1,197406178	0,595056151
Q71LE2	[42-50]	[R].YRPGTVALR.[E]	-0,069758199	0,088547255
A1E295	[129-141]	[R].VNVEVSAEDMLTC.[C]	0	NaN
Q28970	[2-15]	[M].VILQQGDYVWMDLR.[S]	0	NaN
C0HL13	[2398-2430]	LHFDPEDYNVPFTAISVEETAVAVDYDSIDN	0	NaN
P01965	[1-11]	[-].VLSAADKANVK.[A]	-1,163160499	0,404023063
O62839	[434-444]	[N].SANEDNVTQVR.[T]	0	NaN
A1XQR6	[2-18]	[M].PVAVGPYGQSQPSCFDR.[V]	-0,350170001	0,635944036
P80031	[1-11]	[-].PPYTITYFPVR.[G]	0,19274724	0,241069812
P00889	[27-47]	[A].SASSTNLKDILADLIPKEQAR.[I]	0,458651682	0,593708109
P80928	[1-12]	[-].MPMFVVNTNVPR.[A]	-0,03191246	0,042399136
Q9MYT8	[1-15]	[-].MVPPVQVSPLIKLGR.[Y]	-0,254668001	0,394290825
P02067	[1-31]	MVHLSAEEKEAVLGLWGKVNVDEVGGEALG	0	NaN
Q6QAQ1	[44-62]	[V].MVGMGQKDSYVGDEAQSKR.[G]	1,373175828	0,63368242
Q2XVP4	[203-214]	[F].MVDNEAIYDICR.[R]	-0,046888631	0,026432089
P63053	[1-27]	G].MQIFVKTLTGKTITLEVEPSDTIENVK.[A	0	NaN
P80229	[346-362]	[L].MPEEDFIADHPFIFFIR.[H]	0	NaN
Q9MYT8	[3-15]	[V].PPVQVSPLIKLGR.[Y]	0,230132569	0,49854279
Q4U116	[415-438]	Q].MNGDTPHDGGHGGGGHADCEELQR.[T	0	NaN
P01965	[81-92]	[L].SALSDLHAHKLR.[V]	0	NaN

Q29036	[2-11]	[M].SASVLSVISR.[F]	-0,042673224	0,083797502
Q8WN98	[1-22]	[-].MKDPDPSQVYRPDMDPEAAKDK.[G]	0,224130511	0,123477731
O62839	[410-424]	[F].SAPEQTHSALEHCTR.[Y]	0	NaN
Q04967	[43-51]	[S].YVAFTDTER.[L]	0	NaN
P50441	[70-82]	[Y].NEWDPLEEVIVGR.[A]	0,820659156	0,329362036
Q9MZ16	[124-139]	[V].NLGCDVDFDIAGPSIR.[G]	1,555322612	0,854892425
P62802	[26-36]	[D].NIQGITKPAIR.[R]	0,005712592	0,009656979
P00371	[86-99]	[A].NMGLTPVSGYNLFR.[E]	1,529831876	1,121831913
P02067	[109-117]	[G].NVIVVVLAR.[R]	-0,071189473	0,024621562
P02554	[100-121]	[N].NWAKGHYTEGAELVDSVLDVVR.[K]	0	NaN
Q29594	[2-13]	[M].PFSNSHNTLKLR.[F]	-0,383848576	0,355734855
P80928	[2-12]	[M].PMFVVNTNVPR.[A]	-0,061773945	0,10633391
Q9TSX9	[2-22]	[M].PGGLLLGDEAPNFEANTTIGR.[I]	0,203302078	0,419582115
P81045	[1-16]	[-].PGLAAAIPAPPESQEK.[K]	1,014042935	0,616648609
P47788	[1-25]	-].MKPPAACAGDALDVAAPCSAVNYLR.[W	0	NaN
P15145	[827-840]	[R].SALACSNEVWLLNR.[Y]	0	NaN
Q0QF01	[44-75]	AKVSDAISTQYPVVDHEFDAVVVGAGGAGL	-0,058278843	0,093527356
Q2XQV4	[22-55]	APTQAVPAPNQQPEIFYNQIFINNEWHDAI	0	NaN
P01965	[1-11]	[-].VLSAADKANVK.[A]	-1,408785701	0,908136817
O02713	[52-61]	[R].SPGVAELSQR.[C]	-0,062488802	0,104640032
Q9GMB0	[26-39]	[A].SPEALPLVNEDVKR.[T]	0,135309344	0,250803062
P08835	[89-105]	[K].SIHTLFGDKLCAIPSLR.[E]	-1,104969919	0,497075251
O62839	[395-424]	QDNQGGAPNYYPNSFSAPEQTHSALEHCTR	0	NaN
P41367	[23-53]	QHTKAVPQCEPGSGFSFELTEQQKEFQATA	1,339412258	1,169626439
P41367	[23-53]	QHTKAVPQCEPGSGFSFELTEQQKEFQATA	-0,310711342	0,363787874
P41367	[23-46]	[S].QHTKAVPQCEPGSGFSFELTEQQK.[E]	0	NaN
P41367	[23-46]	[S].QHTKAVPQCEPGSGFSFELTEQQK.[E]	0	NaN
Q95334	[65-79]	[D].QGVCPASEDESGNWR.[D]	0	NaN
O62839	[395-424]	QDNQGGAPNYYPNSFSAPEQTHSALEHCTR	0	NaN
Q29201	[2-15]	[M].PSKGPLQSVQVFGR.[K]	-0,036886033	0,040829925
O62839	[395-424]	QDNQGGAPNYYPNSFSAPEQTHSALEHCTR	0	NaN
A5PF10	[386-398]	[D].QAPQLYVLYEKGR.[N]	1,240827528	0,96909493
P50441	[40-62]	[T].QAATASSGNSCAADDKATDPLPK.[D]	0	NaN

P50441	[40-62]	[T].QAATASSGNSCAADDKATDPLPK.[D]	-0,691121813	0,983758844
P50441	[40-55]	[T].QAATASSGNSCAADDK.[A]	0	NaN
P50441	[40-55]	[T].QAATASSGNSCAADDK.[A]	0	NaN
P05027	[84-107]	[S].QKTEISFRPNDPQSYESYVVSIVR.[F]	0	NaN
P80021	[44-58]	[L].QKTGTAEVSSILEER.[I]	0,05082404	0,073149463
Q29095	[29-40]	[L].QPNFQEDKFLGR.[W]	1,435292857	0,717265195
Q5S1U1	[177-190]	[T].QSAEITIPVTFEAR.[A]	-0,201459168	0,50797491
Q08094	[257-266]	[N].QSGQVFGLGR.[Q]	-0,559481141	0,746991359
Q29221	[240-259]	[Y].QTAISENYQTMSDTTFKALR.[R]	0	NaN
Q2XVP4	[176-214]	TAVVEPYNSILTTHTTLEHSDCAFMVDNEAIY	0	NaN
P01846	[87-105]	[C].QVTHEGTIVEKTVTPSECA.[-]	0	NaN
P80021	[186-204]	[V].REPMQTGIKAVDSLVPIGR.[G]	-1,100989283	0,426549998
P03974	[625-638]	[N].RPDIIDPAILRPGR.[L]	0	NaN
Q9GMB0	[26-38]	[A].SPEALPLVNEDVK.[R]	-0,521784108	0,231799629
P16276	[637-648]	[V].TQEFGPVPDTAR.[Y]	0,011502412	0,005258725
Q6QAQ1	[269-290]	[G].MESCGIHETTFNSIMKCDVDIR.[K]	0	NaN
P00346	[213-229]	[C].TPKVDFPQDQLSTLTGR.[I]	0,997987151	0,563357832
Q52NJ6	[97-110]	[R].STYNHLSSWLTDAR.[N]	-0,566252735	0,19765022
P04366	[13-24]	[N].DIQVQENFDLSR.[I]	1,67289295	1,184869114
C0HL13	[4485-4499]	[M].DIGVSGFGPESAIDR.[S]	1,617111281	1,02431457
A1XQS5	[62-68]	[R].DIDTAAK.[F]	-0,305517445	1,170006369
Q29092	[22-37]	[A].EDEVDVDGTVEEDLGK.[S]	-0,186430333	0,410618749
Q6QAQ1	[4-28]	D].DIAALVVDNGSGMCKAGFAGDDAPR.[A	0,994956428	0,541007196
P26044	[490-509]	[H].DENNAEASAELSNDGVMNHR.[S]	0	NaN
P50828	[27-39]	[L].SLTAGPKHGAEGR.[N]	-1,721249211	1,107670577
Q9TSX9	[9-22]	[G].DEAPNFEANTTIGR.[I]	-0,190515856	0,440497233
P24854	[23-38]	[G].DEAIHCPPCSEEKLAR.[C]	0,079109392	0,125476784
Q9N0F1	[69-89]	[K].DDVITVKTPAFAESVTEGDVR.[W]	-1,259000372	0,535005925
P50441	[224-235]	[Y].DQDYPIYSVEDR.[H]	0	NaN
P48819	[20-27]	[A].DQESCKGR.[C]	0,315963668	0,128904251
P22412	[17-25]	[A].DQFRDLAVR.[I]	0,063083162	0,080300194
P02543	[51-64]	[R].SLYTSSPGGVYATR.[S]	-1,077352546	0,449976705
P02067	[95-105]	[C].DQLHVDPENFR.[L]	-0,040219394	0,035862275

P80021	[197-204]	[V].DSLVPIGR.[G]	-1,589543605	1,289371112
Q9TV61	[1202-1216]	[A].DSVAELGEQIDNLQR.[V]	0	NaN
Q8HZK3	[1332-1339]	[E].DTLSLHIR.[A]	0	NaN
P08835	[25-34]	[R].DTYKSEIAHR.[F]	0	NaN
P08835	[25-34]	[R].DTYKSEIAHR.[F]	0,059071958	0,026266884
P81405	[2-19]	[G].DVCQDCIQMVTDLQNAVR.[T]	0	NaN
P81405	[2-19]	[G].DVCQDCIQMVTDLQNAVR.[T]	-0,686229015	0,331857044
Q9MZ16	[128-139]	[C].DVDFDIAGPSIR.[G]	-0,446536662	0,208817661
P50828	[132-152]	[R].SLQEEFPGVPSPLDAAVECHR.[G]	0	NaN
P02543	[430-440]	[E].SLPLVDTHSKR.[T]	-1,037964302	0,573635588
I3LMB3	[21-37]	[A].EAPQEHDPFTYDYQSLR.[I]	-0,254914338	0,317405096
P80021	[335-345]	[R].EAYPGDVFYLH.[S]	0	NaN
Q5S3G4	[31-49]	[S].MASGGGVPTDEEQATGLER.[E]	-0,55146906	0,225964935
P06867	[20-38]	[G].DSLDDYVNTQGAFLFSLSR.[K]	0	NaN
P16276	[634-648]	[R].NAVTQEFGPVPDTAR.[Y]	0	NaN
C0HL13	[4036-4044]	[R].SMSEHYGER.[C]	-0,192762932	0,394633851
B3SP85	[54-72]	[S].DPPPVNVNLYYESLCNGCR.[Y]	-1,085771592	0,4315181
P48819	[412-427]	[C].EPIQSVYFFSGEEYYR.[V]	0,011928104	0,015287191
P02554	[3-14]	[R].EIVHIQAGQCGN.[Q]	0	NaN
Q6QAQ1	[241-254]	[Y].ELPDGQVITIGNER.[F]	-0,274305393	0,212428812
P02067	[91-105]	[S].ELHCDQLHVDPENFR.[L]	0,274518018	0,170483384
P29700	[17-25]	[V].PHGPILGYR.[E]	0,418075045	0,253246559
C0HL13	[2335-2362]	].SPAEVNYNPCLQNNGGCTHFCFALPQLR.[	0	NaN
P37111	[9-17]	[G].EHPSVTLFR.[Q]	-0,707010563	0,292174168
P00348	[146-162]	[A].SNTSSLQITSLANATTR.[Q]	0,150686151	0,070326141
A5GFQ5	[46-52]	[A].EETDWVR.[L]	-0,142429684	0,334473752
P79263	[807-829]	[K].ETLYSVMPGLKITMDKAGLLLLS.[S]	0	NaN
Q29092	[24-39]	[D].EVDVDGTVEEDLGKSR.[E]	0,030133657	0,016733944
Q29122	[506-522]	[N].EVHYVDNQDCIDLIEAR.[L]	0,826821341	0,409276486
Q29183	[23-39]	[G].EYVGLSANQCAVPAKDR.[V]	1,018772289	0,684784866
P01025	[23-35]	[G].DPIYTIITPNVLR.[L]	-1,19641002	0,75965024
Q6QAQ1	[21-28]	[G].FAGDDAPR.[A]	-0,203304738	0,295676172
P29804	[29-38]	[T].FANDATFEIK.[K]	-0,177913761	0,039134964

P29804	[29-44]	[T].FANDATFEIKKCDLHR.[L]	-0,183353453	0,184586279
P62936	[8-19]	[F].FDIAVDGEPLGR.[V]	1,186171348	0,454422513
C0HL13	[4592-4605]	[N].FENPIYAETENEPK.[V]	0	NaN
Q0QF01	[33-42]	[S].FHFTVDGNKR.[S]	-0,614438246	1,212247102
Q3ZD69	[282-296]	[N].SNLVGAAHEELQQSR.[I]	0	NaN
Q711S8	[24-35]	[G].FPVYDYDPSSLR.[E]	-0,456374103	1,071541557
Q9XT00	[70-81]	[A].FQADVSEAETAR.[R]	0	NaN
Q29095	[32-40]	[N].FQEDKFLGR.[W]	-0,632987053	0,825160537
Q6ITQ4	[92-108]	[R].SNEHENAYENTSEEEGR.[V]	0	NaN
P01025	[958-977]	[R].EEIPPADLSDQVPDTESETK.[I]	0	NaN
Q29092	[22-39]	[A].EDEVDVDGTVEEDLGKSR.[E]	0,015930636	0,017356147
A1XQS5	[62-99]	TAAKFIGAGAATVGVAGSGAGIGTVFGSLIIG	0	NaN
Q2EN81	[24-40]	[P].FAKLVRPPVQIYGIEGR.[Y]	0,0070301	0,011616397
Q007T0	[31-38]	[Q].TAAATAPR.[I]	-0,582679983	1,14398374
Q95312	[41-56]	[F].TFASPTQVFFNGANVR.[Q]	-0,095570243	0,120561597
Q70BM6	[2-9]	[M].TFAELVDR.[V]	0,000767345	0,001102019
P00258	[59-72]	[W].SSSEDKITVHFINR.[D]	-0,305118173	0,113428913
Q2XVP4	[187-214]	N].SILTTHTTLEHSDCAFMVDNEAIYDICR.[R	0	NaN
Q2XVP4	[187-214]	N].SILTTHTTLEHSDCAFMVDNEAIYDICR.[R	0	NaN
Q3ZD69	[428-435]	[R].SSFSQHAR.[T]	0	NaN
Q0QF01	[43-75]	AKVSDAISTQYPVVDHEFDAVVVGAGGAGI	0,058785323	0,071976154
F1RKQ4	[9-35]	].SVAGCADDALAGLVACNPSLQLLQGHR.[	0	NaN
P15145	[573-599]	].SSAFDYLWIVPISSIKNGVMQDHYWLR.[D	0	NaN
P50441	[270-282]	[R].SQVTNYMGIEWMR.[K]	0,283187141	0,205640978
P16276	[412-424]	[K].SQFTITPGSEQIR.[A]	-0,426057819	0,697131843
P10173	[2-7]	[A].SQDSFR.[I]	-0,282144733	0,905536054
P80147	[29-54]	I].SQAAAKVDVEFDYDGPLMKTEVPGPR.[S	-0,773177618	0,281511166
P04366	[5-24]	[A].SPVLTLPNDIQVQENFDLSR.[I]	-0,739249562	0,3789151
P01846	[80-105]	[K].SSSGFTCQVTHEGTIVEKTVTPSECA.[-]	0	NaN
Q29307	[26-34]	[F].SSDTPEGVR.[S]	-0,906062248	0,463963485
P80147	[230-250]	[H].SKAIHKIDIPSFDWPIAPFPR.[L]	0	NaN
C0HL13	[641-655]	[R].SSTRPFGVTVYHAIR.[Q]	-0,256691525	0,102396954
P24964	[64-71]	[F].SSVTHICR.[D]	1,099776966	0,552222322

P00506	[30-52]	[A].SSWWAHVEMGPPDPILGVTEAFK.[R]	0	NaN
P00506	[30-53]	[A].SSWWAHVEMGPPDPILGVTEAFKR.[D]	0	NaN
P00506	[30-53]	[A].SSWWAHVEMGPPDPILGVTEAFKR.[D]	-0,269904373	0,586265764
Q02038	[38-45]	[M].SSYTVDGR.[N]	-0,017897501	0,028668421
Q8SQ26	[7-22]	[S].STADGVAVVGSGLIGR.[S]	0,966789368	0,447685442
P50441	[38-55]	[Q].STQAATASSGNSCAADDK.[A]	0	NaN
P50441	[38-55]	[Q].STQAATASSGNSCAADDK.[A]	0	NaN
P50441	[38-62]	[Q].STQAATASSGNSCAADDKATDPLPK.[D]	-0,087177108	0,056546063
P50441	[38-62]	[Q].STQAATASSGNSCAADDKATDPLPK.[D]	0,875029271	0,556334826
Q767M3	[29-41]	[L].STQSEPHGSPISR.[R]	0	NaN
O19069	[279-295]	[K].SKPVVSFIAGLTAPPGR.[R]	0	NaN
Q6QAQ1	[232-254]	[A].SSSSLEKSYELPDGQVITIGNER.[F]	0,17364755	0,109033456
P09571	[259-280]	[R].SVDGQEDSIWELLNQAQEHFGR.[D]	0	NaN
Q29318	[40-55]	[H].SVGICGSDVHYWQHGR.[I]	0	NaN
P33198	[301-322]	[T].SVLVCPDGKTIEAEAAHGTVTR.[H]	0	NaN
Q6QAQ1	[106-116]	[L].TEAPLNPKANR.[E]	0,013349237	0,014686636
C0HL13	[680-688]	[R].TDNGGLGYR.[C]	-0,284388104	0,833212309
P08835	[76-105]	].TCVADESAENCDKSIHTLFGDKLCAIPSLR.[	0	NaN
P50828	[29-39]	[L].TAGPKHGAEGR.[N]	-1,50848949	0,958448525
Q6QAQ1	[52-62]	[D].SYVGDEAQSKR.[G]	-0,358727573	0,485250693
Q0QF01	[196-207]	[R].TGHSLLHTLYGR.[S]	0	NaN
Q6QAQ1	[239-254]	[K].SYELPDGQVITIGNER.[F]	-0,217345828	0,63243242
Q8MIR4	[394-422]	].SYAPGGPAYQPVVEAFGTDILHKDGTINR.[	0	NaN
P00506	[31-53]	[S].SWWAHVEMGPPDPILGVTEAFKR.[D]	0	NaN
P01025	[670-677]	[R].SVQLMEKR.[M]	-0,765768111	0,387978439
P79263	[695-707]	[L].SVPDETSHDMDSR.[I]	0	NaN
O62839	[445-456]	[R].TFYLNVLNEEER.[K]	0	NaN
P62895	[29-39]	[K].TGPNLHGLFGR.[K]	0,07840553	0,075647931
P00371	[87-99]	[N].MGLTPVSGYNLFR.[E]	-1,199353397	0,395463479
P79273	[27-42]	[H].TIFQSVELPETYQMLR.[Q]	0	NaN
C0HL13	[3192-3203]	[N].TNIEPYLIFSNR.[Y]	0,325613041	0,179331927
C0HL13	[4590-4605]	[N].TNFENPIYAETENEPK.[V]	0	NaN
P02543	[101-113]	[R].TNEKVELQELNDR.[F]	0,970956681	0,387059977

Q29099	[109-122]	[N].TMVNYYTSVTPVLR.[G]	0	NaN
P80031	[4-11]	[Y].TITYFPVR.[G]	-1,244892009	1,185152321
P04366	[9-24]	[L].TLPNDIQVQENFDLSR.[I]	-1,067929675	1,025123698
P08835	[92-105]	[H].TLFGDKLCAIPSLR.[E]	-0,732860334	0,295183084
P80021	[46-58]	[K].TGTAEVSSILEER.[I]	-0,099955332	0,154288421
P33198	[310-322]	[K].TIEAEAAHGTVTR.[H]	0	NaN
Q2EN81	[77-85]	[A].SIMNPYVKR.[S]	0,621015532	0,208467478
Q29261	[2-23]	[M].TKLNAQVKGSLNVTTPGVQIWR.[I]	0,154024065	0,173453721
P08835	[435-451]	[Y].TKKVPQVSTPTLVEVAR.[K]	-0,966084965	0,432809493
P28839	[1-9]	[-].TKGLVLGIY.[S]	-0,334148226	0,14451993
P41367	[25-53]	].TKAVPQCEPGSGFSFELTEQQKEFQATAR.	0,95144687	0,563814829
Q29261	[757-788]	TNLSSGPLPIFPLEQLVNKPAEELPQGVDPSF	0	NaN
P26044	[487-509]	[H].DEHDENNAEASAELSNDGVMNHR.[S]	0	NaN

The difference indicates doxycycline vs. control group. Proteins were considered significant if -log10 (p-value) > 1.3. Significant p-values are marked bold.

Abbreviations: N/A = not available
