# Supplementary material for: Doxycycline Alters the Porcine Renal Proteome and Degradome during Hypothermic Machine Perfusion
Source: Curr Issues Mol Biol. 2022 Jan 23;44(2):559–77. doi: 10.3390/cimb44020039 (PMC8928973; doi:10.3390/cimb44020039)
Supplement: Supplementary file 1 [file cimb-44-00039-s001.zip › Table S3.pdf]

Table S3. Identified degradation products post-reperfusion (T240).

Accession number	Position in protein	Protein substrate	Difference (DOXY vs Control)	-Log10 (P-value)
Q4KRV1	[27-39]	ASQIEDQAEQFFR	-1,46961775	**1,344632291**
P06348	[68-82]	ALAAAGYDVEKNNSR	-1,834505811	**1,937166701**
Q6PQZ1	[237-243]	SSDLTDR	-1,627484472	**1,308548311**
Q9GMB0	[26-38]	SPEALPLVNEDVK	1,361178837	**1,42029195**
P50441	[400-413]	SLGGGFHCWTCDVR	2,363029148	**2,1979764**
P80021	[254-262]	STVAQLVKR	2,595956121	**2,66038676**
P01965	[1-11]	VLSAADKANVK	3,872360738	**1,853148995**
P02067	[4-31]	EEKEAVLGLWGKVNVDEVGGEA	2,976594205	**2,549326958**
P20305	[426-441]	QHGMDDDGTGQKQIWR	2,544495525	**2,858050497**
Q71LE2	[19-27]	KQLATKAAR	2,545870657	**2,475343653**
O02705	[603-613]	STYGWTANMER	2,326072868	**2,291372547**
P01965	[1-9]	VLSAADKAN	3,335860627	**2,458842047**
Q9GMB0	[26-39]	SPEALPLVNEDVKR	2,553414504	**2,658335044**
P14477	[1-13]	AIDYDEDEDGRPK	1,989643943	**1,869734139**
Q9MZ15	[278-294]	SINAGGHKLGLALELEA	2,237813571	**2,51187527**
P02543	[430-440]	SLPLVDTHSKR	1,29316403	**1,482414286**
Q007T0	[31-38]	TAAATAPR	2,688556539	**1,402358045**
P80021	[447-463]	AQFGSDLDAATQQLLSR	2,124042105	**1,637939485**
P62279	[5-19]	HAPGKGLSQSALPYR	2,109199984	**2,227954405**
Q6QAQ1	[53-62]	YVGDEAQSKR	1,688549853	**1,893923559**
P81140	[363-372]	GISDEYHVIR	1,630846342	**2,117351532**
P04366	[5-24]	SPVLTLPNDIQVQENFDLSR	2,201940713	**1,589958873**
P00503	[2-32]	[illegible]VFAEVPQAQPVLVFKLIADFREDI[illegible]	2,971584392	**2,666090315**
Q6QAQ1	[234-254]	SSLEKSYELPDGQVITIGNER	2,591555225	**2,626028358**
P00348	[149-162]	SSLQITSLANATTR	2,522485167	**2,438963471**
Q29307	[26-34]	SSDTPEGVR	1,437123686	**1,880169569**
P80021	[186-204]	REPMQTGIKAVDSLVPIGR	2,631893034	**1,319978006**
Q29550	[19-33]	GQPASPPVVDTAQGR	1,709115875	**1,952636149**
O19069	[143-154]	ITEGIPQQDMVR	2,381444872	**2,344821579**
P28839	[1-9]	TKGLVLGIY	2,347059597	**1,587392487**
P41367	[380-392]	GFNTEYPVEKLMR	2,303338655	**2,00401483**

P80021	[46-58]	TGTAEVSSILEER	0,758423628	**1,531676288**
Q6QAQ1	[273-290]	GIHETTFNSIMKCDVDIR	1,327505804	**1,299676068**
P01965	[1-8]	VLSAADKA	3,239788332	**3,018813202**
P01965	[12-31]	AAWGKVGGQAGAHGAEALER	0,98314961	**1,861941283**
Q29371	[43-53]	APPTAYIDFAR	1,320549128	**1,571370447**
P62936	[9-19]	DIAVDGEPLGR	2,598131984	**2,375747488**
P80031	[3-11]	YTITYFPVR	1,725892376	**2,116568107**
P08059	[107-124]	SNTPILVDGKDVMPEVNR	2,366774738	**2,030453992**
Q0QF01	[199-207]	SLLHTLYGR	2,136529182	**2,095965321**
P28491	[21-36]	IYFKEQFLDGDGWTDR	1,749788404	**1,837288915**
Q8MJ39	[26-37]	LESYEINPFLNR	2,287093584	**1,921396722**
P01965	[1-9]	VLSAADKAN	3,198535463	**2,632968621**
P53590	[258-270]	DAKINFDDNAEFR	2,605868523	**2,755583414**
P00348	[148-162]	TSSLQITSLANATTR	2,014008999	**1,348922235**
P01025	[747-762]	SDLDEEIIPEEDIISR	0,859011505	**1,421924759**
P80928	[5-12]	VVNTNVPR	1,859319517	**2,323060853**
P00346	[27-52]	'AVLGASGGIGQPLSLLLKNSPLVS	4,113600236	**2,678584029**
Q2QLE2	[2-21]	GLETEKADVQLFMDDDSYSR	2,533496167	**2,585528534**
P02067	[2-9]	VHLSAEEK	3,973790524	**1,920970757**
Q4FAT7	[23-35]	LLQGGMLYPQESR	2,07619197	**1,415638192**
P62802	[25-36]	DNIQGITKPAIR	2,25487199	**2,258203525**
Q2XVP4	[54-64]	SETGAGKHVPR	2,251696873	**1,299501008**
P51779	[27-36]	ILGGQEAKSH	2,34062529	**2,333682591**
Q95339	[4-10]	VVPLKDR	2,497631814	**2,645530521**
P82460	[2-21]	VKQIESKYAFQEALNSAGEK	2,617997425	**3,099188913**
Q0MVN8	[188-201]	IVLQNGAHEVFNHR	1,657768931	**1,574605874**
P50828	[24-39]	HPLSLTAGPKHGAEGR	0,670712831	**1,443406966**
Q2F7Z7	[36-50]	EAFDSVLGDTASCHR	2,634973983	**2,994494827**
P79384	[30-38]	TQPVSVNER	0,719865195	1,734355062
Q0MVN8	[193-201]	GAHEVFNHR	0	NaN
P00889	[28-47]	ASSTNLKDILADLIPKEQAR	1,420966715	0,962985232
P23695	[25-48]	AYRPSETLCGGELVDTLQFVCGDF	0	NaN
Q07717	[83-100]	LLVHTEFTPNAVDQYSCR	0	NaN

P80229	[264-275]	LELLEVNVHLPR	1,025953666	0,90730132
Q19KI0	[2-8]	AWNTNLR	-1,331264745	1,147343117
P50441	[42-62]	ATASSGNSCAADDKATDPLPK	0	NaN
Q95339	[2-10]	ASVVPLKDR	-0,031595551	0,042222188
P29700	[294-304]	AVPPGIPPVHR	0	NaN
P41367	[24-53]	AVPQCEPGSGFSFELTEQQKEFQA	-0,183630282	0,054865641
A1XQT2	[2-12]	ATSSLTKPQMR	0,075164006	0,058410155
Q5S1U1	[29-38]	LFDQAFGLPR	-0,230243177	0,077778108
Q29550	[15-33]	ATWAGQPASPPVVDTAQGR	0	NaN
P01965	[13-31]	AWGKVGGQAGAHGAEALER	0,043703383	0,032730858
Q6QAQ1	[29-37]	AVFPSIVGR	1,602475863	1,037308424
P27917	[21-41]	IEAEDTSLLDKMQDYVKQATR	0,207257027	0,228740308
P06348	[69-82]	LAAAGYDVEKNNSR	1,396938081	0,755282836
P80021	[135-149]	GAIVDVPVGEELLGR	0,831711548	0,269286879
Q71LE2	[104-117]	LFEDTNLCAIHAKR	1,479276559	0,924818896
Q8WN93	[23-39]	ETEEENPDDLIQLTVTR	0	NaN
Q9MYY8	[3-33]	ELPEMYDYDLIIIGGGSGGLAAAKE	0	NaN
Q6Q7J2	[319-328]	IIIPQNQVNR	1,450342144	0,434172403
P00889	[26-47]	ASASSTNLKDILADLIPKEQAR	0,156231206	0,154135216
Q6QAQ1	[231-254]	ASSSSLEKSYELPDGQVITIGNER	1,746545315	0,778931149
P51779	[27-40]	ILGGQEAKSHERPY	0,768577156	0,320496348
P51779	[27-38]	ILGGQEAKSHER	0,254348486	0,292709772
C0HL13	[2757-2773]	CDHYNDCGDNSDESGCR	0	NaN
Q75NG9	[87-95]	LIDSHFEAR	0	NaN
A5GZW8	[57-70]	ASSKAASLHWTGER	0,037379736	0,049265304
P79273	[25-42]	LHTIFQSVELPETYQMLR	0	NaN
Q29268	[2-20]	APSGLKAVVGEKILSGVIR	-0,068566733	0,024278472
P10173	[1-7]	ASQDSFR	-0,046928885	0,088276412
Q71LE2	[96-117]	ASEAYLVGLFEDTNLCAIHAKR	0	NaN
P79273	[25-42]	LHTIFQSVELPETYQMLR	0	NaN
P12037	[51-66]	ARFAQEAEAARVELQK	0,117486182	0,212083783
P00503	[2-26]	APPSVFAEVPQAQPVLVFKLIADFR	0,295379724	0,557849484
P79273	[25-42]	LHTIFQSVELPETYQMLR	0	NaN

Q007T0	[29-38]	AQTAAATAPR	0,547553692	0,396872677
P79273	[25-38]	LHTIFQSVELPETY	0	NaN
Q28943	[2-21]	APVLSKDVADIESILALNPR	0,786640964	0,361628402
Q4FAT7	[23-35]	LLQGGMLYPQESR	-0,309726172	0,17309858
Q95332	[2-8]	APVGDKK	2,071187651	1,079192584
D0VWV4	[30-40]	LGTTAKEEMER	0,060090905	0,063123682
Q5S3G4	[32-49]	ASGGGVPTDEEQATGLER	0,18846689	0,357263568
P08835	[86-105]	CDKSIHTLFGDKLCAIPSLR	0	NaN
P02067	[6-31]	EKEAVLGLWGKVNVDEVGGEAL	2,198192622	0,96701244
Q6QAQ1	[17-28]	CKAGFAGDDAPR	0	NaN
P80229	[265-275]	ELLEVNVHLPR	-0,180081691	0,075598263
Q6QAQ1	[269-290]	MESCGIHETTFNSIMKCDVDIR	0	NaN
P28491	[18-36]	EPTIYFKEQFLDGDGWTDR	0,061306888	0,065551714
P48819	[412-427]	EPIQSVYFFSGEEYYR	1,012392842	0,513942858
P04366	[18-24]	ENFDLSR	-0,194943418	0,067873225
P02554	[3-14]	EIVHIQAGQCGN	0	NaN
Q6QAQ1	[241-254]	ELPDGQVITIGNER	0,784089625	1,158025085
P02067	[91-105]	ELHCDQLHVDPENFR	-0,450011766	0,171645306
P27917	[24-41]	EDTSLLDKMQDYVKQATR	1,511849791	0,852572838
Q71LE2	[74-84]	EIAQDFKTDLR	-0,10454254	0,032198899
Q7M329	[159-173]	EKVQTLGQIELCLTR	1,317688338	0,785773542
P37111	[9-17]	EHPSVTLFR	1,000645111	0,758413114
P50447	[25-39]	EGLQGHAVQETDVPR	0,140349414	0,231348335
P37111	[7-17]	EGEHPSVTLFR	1,077624963	0,605750559
A5GFQ5	[46-52]	EETDWVR	-0,150292554	0,220032339
P79263	[807-829]	ETLYSVMPGLKITMDKAGLLLLS	0	NaN
Q29092	[24-39]	EVDVDGTVEEDLGKSR	-0,320276041	0,125652797
P15145	[895-904]	FSNLIQGVTR	-0,026256781	0,029291063
Q29095	[32-40]	FQEDKFLGR	-0,035846662	0,009576032
Q9XT00	[70-81]	FQADVSEAETAR	0	NaN
Q711S8	[24-35]	FPVYDYDPSSLR	0,401645166	0,529249385
P04366	[30-43]	FHVAVGSTCPWLKR	0,718542695	0,223582134
Q0QF01	[33-42]	FHFTVDGNKR	0,1767592	0,275631306

P80021	[61-73]	GADTSVDLEETGR	0	NaN
C0HL13	[4592-4605]	FENPIYAETENEPK	0	NaN
P29804	[29-44]	FANDATFEIKKCDLHR	0,238935127	0,134997578
P29804	[29-38]	FANDATFEIK	-0,135869773	0,036878617
Q2EN81	[24-40]	FAKLVRPPVQIYGIEGR	0,060878791	0,105625002
Q6QAQ1	[21-28]	FAGDDAPR	1,625187021	0,963285731
Q29183	[23-39]	EYVGLSANQCAVPAKDR	-0,654377705	0,312595337
Q29122	[506-522]	EVHYVDNQDCIDLIEAR	0,103513687	0,038078029
P62936	[8-19]	FDIAVDGEPLGR	-0,019980535	0,007279731
P63221	[4-15]	DAGEFVDLYVPR	1,377450309	0,736436405
C0HL13	[3938-3966]	QHLVCDDVDDCGDHFDETGCNTC	0	NaN
P14477	[1-19]	AIDYDEDEDGRPKVHVDAR	1,775955158	1,021602569
P01965	[12-31]	AAWGKVGGQAGAHGAEALER	0	NaN
Q6QAQ1	[295-312]	ANTVLSGGTTMYPGIADR	0	NaN
P50441	[52-82]	KATDPLPKDCPVSSYNEWDPLEEV	0	NaN
P79382	[2-9]	ADLTELMK	0,175359498	0,061954683
P80021	[62-73]	ADTSVDLEETGR	-0,235930753	0,249608413
A1Z623	[31-39]	AEFSSESCR	0,355510219	0,557018574
P00371	[84-99]	AANMGLTPVSGYNLFR	0	NaN
Q8SPS7	[19-41]	AETGNEATDATDDSCPKPPEIPK	0	NaN
P00346	[66-74]	ADLSHIETR	1,282024604	0,665403266
O02705	[599-613]	CIVTSTYGWTANMER	0	NaN
O62839	[411-424]	APEQTHSALEHCTR	0	NaN
P81405	[7-19]	CIQMVTDLQNAVR	-0,338762165	0,120088396
C0HL13	[3645-3675]	WKCDVDNDCGDYSDEPLQECMC	0	NaN
Q6QAQ1	[17-28]	CKAGFAGDDAPR	0,308717508	0,432128865
Q8SPS7	[19-41]	AETGNEATDATDDSCPKPPEIPK	-0,592791182	0,280685257
C0HL13	[3951-3966]	CGDHFDETGCNTGEER	0	NaN
P06348	[71-82]	AAGYDVEKNNSR	0,236901441	0,395676981
P80147	[31-54]	AAAKVDVEFDYDGPLMKTEVPGP	1,751870636	1,037715025
Q0QF01	[45-75]	DAISTQYPVVDHEFDAVVVGAGG	0	NaN
Q0QF01	[45-75]	DAISTQYPVVDHEFDAVVVGAGG	-1,352118926	1,144183358
P00346	[25-52]	KVAVLGASGGIGQPLSLLLKNSPLV	1,050496593	0,621614344

Q2EN81	[25-40]	AKLVRPPVQIYGIEGR	-0,034663801	0,034709611
Q06AU5	[2-24]	\GGGAGDPGQGAAAAAAAAPET	0	NaN
Q6QAQ1	[19-28]	AGFAGDDAPR	0,323503282	0,563949175
P50441	[51-82]	KATDPLPKDCPVSSYNEWDPLEE	0,218879904	0,08004153
Q04967	[225-238];A5A8V7	AGDTHLGGEDFDNR	-1,278132409	1,10698603
P50390	[23-41]	AGAGESKCPLMVKVLDAVR	-0,727960248	0,226050794
Q8MK48	[2-15]	AFNDLLLQVGGVGR	0,499110782	1,120106269
P14460	[1-17]	AEVQDKGEFLAEGGGVR	1,617061606	0,848263432
Q8SPS7	[19-49]	NEATDATDDSCPKPPEIPKGYVEH	0	NaN
Q8SPS7	[19-49]	NEATDATDDSCPKPPEIPKGYVEH	0,766847628	0,169668709
Q8SPS7	[19-49]	NEATDATDDSCPKPPEIPKGYVEH	0	NaN
P47788	[8-25]	AGDALDVAAPCSAVNYLR	0	NaN
P79263	[28-46]	HKNDINIYSLTVDSKVSSR	0,385734036	0,100310267
Q29318	[11-20]	LVVHGPGDLR	0,281731514	0,10975623
P33198	[142-157]	HAHGDQYKATDFVVDR	0,757877876	0,279697794
Q29048	[72-81]	GVSVGDPVLR	-0,044258354	0,016944449
P00571	[116-128]	LYVDAGPETMTKR	-0,340646233	0,146996509
P09571	[455-462]	SCHTAVDR	0	NaN
Q28955	[2-8]	PLLVEGR	1,133592797	0,985107965
Q19KI0	[2-8]	AWNTNLR	0,017431962	0,019156974
A1XQT2	[4-12]	SSLTKPQMR	0,646808872	0,869472431
P16225	[138-143]	FLLSKR	-1,484027573	0,644734074
Q6RVA9	[3-19]	GGKYVDSEGHLYTVPIR	-0,069561507	0,026091078
Q6QAQ1	[89-95]	TFYNELR	-1,600277272	0,950096462
P80021	[448-463]	QFGSDLDAATQQLLSR	-0,003090637	0,001187287
P08835	[25-33]	DTYKSEIAH	1,416500068	0,690759659
P01025	[958-997]	DQVPDTESETKILLQGTPVAQMV	0	NaN
P08835	[27-34]	YKSEIAHR	1,056732171	0,486197947
P80021	[285-301]	LQYLAPYSGCSMGEYFR	0	NaN
P62802	[81-93]	TVTAMDVVYALKR	1,317323625	1,105263617
P00371	[93-99]	SGYNLFR	-0,744076562	0,347122168
P29700	[305-310]	SHYDLR	0,290615693	0,078791047
P05027	[176-182]	VIIKLNR	0,031912683	0,010100025

P01965	[15-31]	GKVGGQAGAHGAEALER	0	NaN
P29797	[2-13]	GCLGNSKTEDQR	-0,05538417	0,023076172
P14460	[9-17]	FLAEGGGVR	2,324737784	1,062202852
Q4U116	[336-342]	SYHEIGR	0,274071824	0,694951432
P83662	[1-8]	MKIDIHSH	-1,340080396	1,070056666
P26042	[481-495]	DEQDENGAEASADLR	0	NaN
Q6QAQ1	[178-183]	LDLAGR	-0,224217142	0,098647885
P11708	[143-157]	SAPSIPKENFSCLTR	-0,4531607	0,190423983
Q8WN98	[1-20]	MKDPDPSQVYRPDMDPEAAK	-0,397491402	0,126415698
Q71LE2	[58-64]	STELLIR	0,147486615	0,218731691
P01025	[671-677]	VQLMEKR	-0,051450751	0,013527234
P50441	[39-55]	TQAATASSGNSCAADDK	0	NaN
Q6SEG5	[1-19]	MQLKPMEINPEMLNKVLTR	0	NaN
Q5U9S1	[109-114]	LEGQIR	0	NaN
P50390	[26-41]	GESKCPLMVKVLDAVR	1,483273647	1,059337006
Q1KYT0	[247-253]	AASEFYR	-0,062794383	0,021883406
C0HL13	[3193-3203]	NIEPYLIFSNR	-0,528026089	0,283767816
O97763	[20-25]	EPVHFR	-1,550960526	1,203851326
Q9TSX9	[3-22]	GGLLLGDEAPNFEANTTIGR	0	NaN
Q6QAQ1	[301-312]	GGTTMYPGIADR	-0,237313279	0,10638857
P26044	[2-8]	PKPINVR	1,536289473	0,786286854
A1E295	[78-87]	MILPKSFDAR	0,089480609	0,02373515
P00791	[16-23]	LVKVPLVR	0,457116873	0,136167833
P00636	[144-158]	STDEPSEKDALQPGR	-0,307752799	0,122145102
Q7YS91	[40-60]	TSSATSPSSHLPGSLSELVER	0	NaN
Q29308	[3-16]	GVTVKDVNQQEFVR	-0,473933587	0,280089137
Q71LE2	[107-117]	DTNLCAIHAKR	-0,187278264	0,078896651
P08059	[22-27]	SDLNLR	0,089904234	0,132475729
P80928	[64-74]	SIGKIGGAQNR	0,26483777	0,487481286
P81140	[12-20]	SSRPEFDWR	-0,166855024	0,084909806
P17741	[2-10]	GKGDPNKPR	-1,625856902	1,174220535
P00795	[1-8]	GPIPEVLK	-0,306482443	0,118322162
P62802	[57-68]	GVLKVFLENVIR	-0,071373135	0,027568364

P01025	[967-997]	’DTESETKILLQGTPVAQMVEDAII	0	NaN
P50828	[30-39]	AGPKHGAEGR	-0,471166939	0,167432295
P50441	[38-62]	TQAATASSGNSCAADDKATDPLP	0	NaN
O02668	[68-73]	STITSR	0,387789506	0,80306503
Q66RM2	[1-6]	MNIFDR	0,501750654	0,168921181
Q04967	[39-51];A5A8V7	TTPSYVAFTDTER	0	NaN
Q04967	[68-74];A5A8V7	TVFDAKR	0,960805195	0,260485435
Q95333	[22-29]	HPKSLNQR	0	NaN
P80021	[195-204]	AVDSLVPIGR	0,045361434	0,014462663
Q8WNV7	[21-27]	ASTGVER	-0,470561299	0,226371021
C0HL13	[3068-3078]	EETTCPPHQFR	-0,161612925	0,061222262
C0HL13	[4036-4044]	SMSEHYGER	0	NaN
P01965	[1-7]	VLSAADK	-0,643476119	0,278457027
Q07717	[21-32]	VARPPKVQVYSR	1,606228658	1,098172438
Q29550	[17-33]	WAGQPASPPVVDTAQGR	0	NaN
P02067	[35-41]	VYPWTQR	2,136185337	0,91069826
P29700	[18-25]	HGPILGYR	0,538665427	0,677972942
O11780	[24-38]	GPAKSPYQLVLQHSR	0	NaN
P29700	[335-356]	GAQPSIPAADGSVPVVRPCPGR	0	NaN
P50828	[26-39]	LSLTAGPKHGAEGR	1,417339277	0,725179796
Q29318	[9-20]	LSLVVHGPGDLR	1,170520459	0,513138848
P80147	[28-54]	QAAAKVDVEFDYDGPLMKTEVPG	0,576811117	0,238150836
Q2XVP4	[286-308]	LSVAEITNACFEPANQMVKCDPR	0	NaN
P50828	[28-39]	LTAGPKHGAEGR	0,320205284	0,444245476
Q6QAQ1	[105-116]	LTEAPLNPKANR	0,502940704	0,170564667
Q8WNQ7	[377-386]	LTERPIFYYR	1,288022722	1,257273705
Q9N0F1	[196-222]	SKPVSAVKPTAAPPVAEPGAVKG	1,271412686	0,813498781
Q8SPS7	[103-120]	IMGGSLDAKGSFPWQAKM	0,972379667	0,267122676
Q29092	[647-660]	LVASQYGWSGNMER	0,289026392	0,411051902
P02554	[65-77]	LVDLEPGTMDSVR	1,291463008	0,90865495
Q71LE2	[101-117]	LVGLFEDTNLCAIHAKR	0,328658528	0,149886265
P50828	[254-271]	LVLTALLSDNHGATYAFR	-0,130372668	0,048345469
Q2EN81	[27-40]	LVRPPVQIYGIEGR	1,659043982	1,106342957

Q6QAQ1	[8-28]	LVVDNGSGMCKAGFAGDDAPR	-0,181421816	0,056097874
Q6QAQ1	[8-28]	LVVDNGSGMCKAGFAGDDAPR	0,411900537	0,466744759
Q6QAQ1	[8-28]	LVVDNGSGMCKAGFAGDDAPR	0,155038842	0,083835037
Q0MVN8	[46-55]	GVNPVDTYIR	1,487266327	0,790126106
Q9N0F1	[122-145]	GVIEALLVPDGGKVEGGTPLFTLR	0	NaN
D0VWV4	[31-40]	GTTAKEEMER	0,05948588	0,078356291
Q08092	[209-226]	GTNKGASQAGMTAPGTKR	0,624482478	0,284367889
Q08094	[211-227]	GTNKCASQVGMTAPGTR	-0,211143469	0,085842607
P02067	[84-105]	GTFAKLSELHCDQLHVDPENFR	0	NaN
P02067	[89-105]	LSELHCDQLHVDPENFR	1,351990477	1,194033059
Q7M329	[185-200]	LEICEDGPVFYPPPKE	0	NaN
P02067	[32-41]	LLVVYPWTQR	0,270619699	0,387443599
P53590	[39-47]	LNLQEYQSK	0,245647418	0,416165973
P50441	[218-235]	MADELYDQDYPIYSVEDR	0	NaN
P02067	[15-31]	LWGKVNVDEVGGEALGR	0,765058137	0,218916072
Q29307	[25-34]	FSSDTPEGVR	-1,426061922	1,181604555
P29700	[286-304]	LVVGPMVVAVPPGIPPVHR	-0,385578547	0,173153795
P80021	[47-58]	GTAEVSSILEER	-1,237020766	1,000068003
P81405	[1-19]	GDVCQDCIQMVTDLQNAVR	0,109037345	0,203592684
Q0MVN8	[24-43]	LQSDVAIPIPKDNQVLIKVH	0	NaN
P80021	[408-416]	INVGLSVSR	1,128028831	0,922870695
P05024	[208-225]	GCKVDNSSLTGESEPQTR	1,911128713	0,592157005
P81649	[91-118]	NNCHQSAKPVSLTQCSFTGGNYP	0	NaN
A5GFY8	[450-460]	GAVFRPEVPLR	1,795384524	0,944026799
P01965	[19-31]	GQAGAHGAEALER	-0,487723572	0,184692022
P81405	[1-16]	GDVCQDCIQMVTDLQN	0	NaN
P81405	[1-19]	GDVCQDCIQMVTDLQNAVR	-0,638577734	0,342192493
P81405	[1-19]	GDVCQDCIQMVTDLQNAVR	-0,047380538	0,07442053
O46427	[114-123]	GPYPPSMDWR	-0,005090034	0,006297742
Q6QAQ1	[20-28]	GFAGDDAPR	0,624493064	0,186239001
P50390	[21-41]	GPAGAGESKCPLMVKVLDAVR	1,947766468	0,749950704
P80021	[192-204]	GIKAVDSLVPIGR	0,97634112	0,592686674
Q5S3G4	[35-49]	GGVPTDEEQATGLER	-0,439554005	0,136000407

A1XQU5	[2-17]	GKFMKPGKVVLVLAGR	0,0657093	0,077940853
P36968	[74-89]	GKTEVNYTQLVDLHAR	0	NaN
Q06AU5	[7-24]	GDPGQGAAAAAAAAPETR	-0,567507733	0,239876788
P01965	[15-31]	GKVGGQAGAHGAEALER	0,056999113	0,02134336
Q71LE2	[103-117]	GLFEDTNLCAIHAKR	0	NaN
P53590	[39-56]	LNLQEYQSKKLMSDNGVK	-0,392001148	0,19260312
P04366	[8-24]	LTLPNDIQVQENFDLSR	0,925109667	0,465321197
Q6QAQ1	[13-28]	GSGMCKAGFAGDDAPR	1,50253135	0,3849522
Q4U116	[417-438]	GDTPHDGGHGGGGHADCEELQF	0	NaN
O46427	[293-317]	PYWIVKNSWGPQWGMNGYFLI	0	NaN
Q52NJ4	[2-8]	GLLSILR	0,070706755	0,127897744
P04366	[10-24]	LPNDIQVQENFDLSR	0,321831103	0,183948325
Q9TSX9	[4-22]	GLLLGDEAPNFEANTTIGR	0	NaN
		GLLAQR	0	NaN
Q5S3G4	[34-49]	GGGVPTDEEQATGLER	1,379101531	0,735524914
Q95334	[603-618]	GNAFLKINPDHIGFYR	0,102660657	0,104244022
P80928	[82-87]	GLLAER	0,518461552	0,728989723
P81405	[1-9]	GDVCQDCIQ	0	NaN
Q6QAQ1	[273-290]	GIHETTFNSIMKCDVDIR	0,300752927	0,41690154
C0HL13	[1360-1382]	GGCTHLCIQGPFGAQCECPLGYR	0	NaN
P01025	[958-977]	EEIPPADLSDQVPDTESETK	0	NaN
P26044	[487-509]	DEHDENNAEASAELSNDGVMNH	0	NaN
P18648	[25-33]	DDPQSPWDR	0,666335454	0,843858686
P02067	[1-31]	SAEEKEAVLGLWGKVNVDEVGG	0	NaN
Q9MYT8	[1-15]	MVPPVQVSPLIKLGR	0,389651664	0,734959384
P29700	[291-304]	MVVAVPPGIPPVHR	-1,225965132	0,956237378
P80928	[1-12]	MPMFVVNTNVPR	0,213763202	0,386579026
P00889	[27-47]	SASSTNLKDILADLIPKEQAR	-0,876622973	0,428490235
P80031	[1-11]	PPYTITYFPVR	0,006891886	0,008722058
Q6QAQ1	[44-62]	MVGMGQKDSYVGDEAQSKR	-0,193358187	0,100110251
A1XQR6	[2-18]	PVAVGPYGQSQPSCFDR	0,073450459	0,094505725
P01965	[81-92]	SALSDLHAHKLR	0	NaN
P15145	[827-840]	SALACSNEVWLLNR	0	NaN

Q0QF01	[44-75]	DAISTQYPVVDHEFDAVVVGAG	0,362685835	0,598775516
Q2XQV4	[22-55]	QAVPAPNQQPEIFYNQIFINNEW	0	NaN
Q8MJ14	[7-17]	SAAALAAVAPR	1,074226619	0,53207155
P03974	[625-638]	RPDIIDPAILRPGR	0	NaN
O62839	[434-444]	SANEDNVTQVR	0	NaN
C0HL13	[26-34]	RECLGNEFR	-1,567645422	1,152672895
Q2XVP4	[203-214]	MVDNEAIYDICR	0,225220727	0,081293391
P80229	[346-362]	MPEEDFIADHPFIFFIR	0	NaN
P02067	[109-117]	NVIVVVLAR	1,503971015	0,514122544
P00371	[86-99]	NMGLTPVSGYNLFR	-0,913905543	0,589367494
P62802	[26-36]	NIQGITKPAIR	-0,88098661	0,429157585
Q9MZ16	[124-139]	NLGCDVDFDIAGPSIR	-0,744974644	0,355528689
P13618	[1-9]	NKELDPVQK	-1,437009576	1,063561335
P50441	[70-82]	NEWDPLEEVIVGR	0,34955946	0,151692037
P63053	[1-27]	MQIFVKTLTGKTITLEVEPSDTIENV	0	NaN
Q04967	[43-51];A5A8V7	YVAFTDTER	0	NaN
P0C5I2	[1-13]	MKALILVGGYGTR	1,010658209	0,540218352
Q8WN98	[1-22]	MKDPDPSQVYRPDMDPEAAKDK	-0,204916369	0,082778472
P47788	[1-25]	KPPAACAGDALDVAAPCSAVNY	0	NaN
P04366	[54-62]	MLGEGATER	1,406696801	0,723940154
Q4U116	[415-438]	NGDTPHDGGHGGGGHADCEEL	0	NaN
Q9MYT8	[3-15]	PPVQVSPLIKLGR	0,125669373	0,139360808
P62279	[4-19]	MHAPGKGLSQSALPYR	1,739613232	1,070323689
P02554	[100-121]	NWAKGHYTEGAELVDSVLDVVR	0	NaN
P01846	[87-105]	QVTHEGTIVEKTVTPSECA	0	NaN
Q29221	[240-259]	QTAISENYQTMSDTTFKALR	0	NaN
O62839	[395-424]	QGGAPNYYPNSFSAPEQTHSALE	0	NaN
Q9GMB0	[26-39]	SPEALPLVNEDVKR	0,029704902	0,035021574
O02713	[52-61]	SPGVAELSQR	0,353544591	0,402488376
P01965	[1-11]	VLSAADKANVK	1,479817127	0,757670161
P01965	[1-11]	VLSAADKANVK	1,333528932	0,656173529
P50578	[182-203]	VLQVECHPYLAQNELIAHCQAR	1,323104049	0,700233804
P41367	[23-53]	AVPQCEPGSGFSFELTEQQKEFQ	-0,282288824	0,109586349

Q28970	[2-15]	VILQQGDYVWMDLR	0	NaN
P00355	[2-11]	VKVGVNGFGR	0,064372652	0,08153638
P00355	[2-11]	VKVGVNGFGR	0	NaN
P82460	[2-21]	VKQIESKYAFQEALNSAGEK	-0,268541093	0,105250855
Q06AT9	[2-11]	VKLFIGNLPR	0,184780839	0,278326347
Q07717	[85-100]	VHTEFTPNAVDQYSCR	0	NaN
P02067	[2-31]	AEEKEAVLGLWGKVNVDEVGGE	0,616884536	0,184926105
P00355	[2-11]	VKVGVNGFGR	-0,153637374	0,140139551
Q2XVP4	[176-214]	EPYNSILTTHTTLEHSDCAFMVDN	0	NaN
P41367	[23-53]	AVPQCEPGSGFSFELTEQQKEFQ	0,605274116	0,282547136
P41367	[23-46]	HTKAVPQCEPGSGFSFELTEQQI	0	NaN
Q08094	[257-266]	QSGQVFGLGR	0,193106652	0,623311606
Q5S1U1	[177-190]	QSAEITIPVTFEAR	0,443556302	1,210600941
Q29095	[29-40]	QPNFQEDKFLGR	-0,375224806	0,186234574
P80021	[44-58]	QKTGTAEVSSILEER	-0,095567541	0,081214272
P05027	[84-107]	QKTEISFRPNDPQSYESYVVSIVR	0	NaN
P50441	[40-55]	QAATASSGNSCAADDK	0	NaN
P41367	[23-46]	HTKAVPQCEPGSGFSFELTEQQI	0	NaN
P50441	[40-55]	QAATASSGNSCAADDK	0	NaN
P50441	[40-62]	QAATASSGNSCAADDKATDPLPK	0	NaN
A5PF10	[386-398]	QAPQLYVLYEKGR	-1,344329167	0,833701019
O62839	[395-424]	QGGAPNYYPNSFSAPEQTHSALE	0	NaN
Q29201	[2-15]	PSKGPLQSVQVFGR	0,072506977	0,0794089
O62839	[395-424]	QGGAPNYYPNSFSAPEQTHSALE	0	NaN
Q95334	[65-79]	QGVCPASEDESGNWR	0	NaN
P50441	[40-62]	QAATASSGNSCAADDKATDPLPK	1,932591806	1,262041165
P02067	[2-31]	AEEKEAVLGLWGKVNVDEVGGE	1,38211395	0,634363689
Q29594	[2-13]	PFSNSHNTLKLR	0,189076067	0,284605118
Q9TSX9	[2-22]	PGGLLLGDEAPNFEANTTIGR	0,355338478	0,531275674
Q29195	[79-88]	SCGKDGFHIR	0,6318771	0,270142831
P09571	[116-123]	SCHTGLGR	1,209274121	0,533187128
C0HL13	[345-365]	SCVDFNDCQIWGICDHFCEDR	0	NaN
Q1W0Y2	[17-25]	SHYEEGPGK	-1,543469531	1,103952404

F1RKQ4	[40-48]	SDLDSLKGR	1,325551337	0,515049046
P63053	[57-72]	SDYNIQKESTLHLVLR	-0,104524951	0,093033078
P50441	[49-82]	)KATDPLPKDCPVSSYNEWDPLE	1,755721254	0,609776937
P42174	[1-15]	SEAAADREDDPNFFK	-0,136622554	0,228942538
Q95283	[30-41]	SEDYALPVYVDR	0,345073498	0,74255995
Q9TV69	[27-37]	SEHQVVAVAAR	0,438336833	0,805502468
Q4U116	[1039-1066]	KVPSIKIPMDIMEQQPFLSDSKPS	1,571518387	0,73004684
Q04967	[434-449];A5A8V7	SDNQPGVLIQVYEGER	0,355317912	0,192907616
P80021	[276-301]	ATASDAAPLQYLAPYSGCSMGEYI	0	NaN
Q1W0Y2	[17-29]	SHYEEGPGKNLPF	-0,219805664	0,205408932
Q8WNV7	[258-278]	SEDASYITGETVVVGGGTASR	0	NaN
Q1W0Y2	[17-36]	SHYEEGPGKNLPFSVENKWR	0,587727037	0,280337534
C0HL13	[4633-4647]	SATEDTFKDTANLVR	-0,518744337	0,210644334
O62839	[306-320]	SEYPLIPVGKLVLNR	0,294761784	0,306445994
P80041	[336-347]	SHQGSGLITDYR	0,73460234	1,243753306
P16276	[35-44]	SHFEPHEYIR	0,054625391	0,085695567
P08835	[310-318]	SHCIAEAKR	1,064655398	0,45076872
P26042	[144-156]	SGYLAGDKLLPQR	1,088154261	0,624342724
Q9GJT2	[65-86]	SGYHQAASEHGLVVIAPDTSPR	0	NaN
O19069	[275-295]	SGPKSKPVVSFIAGLTAPPGR	0,633393275	0,213542097
P08835	[29-34]	SEIAHR	2,029910901	0,994475991
Q6QAQ1	[300-312]	SGGTTMYPGIADR	0,696597382	1,066315754
O02705	[675-688]	SGFSLEDPQTHANR	0	NaN
Q1KYT0	[373-400]	ETEDTFIADLVVGLCTGQIKTGAP	1,732724302	0,985534498
C0HL13	[4478-4499]	SGEDVNMDIGVSGFGPESAIDR	0	NaN
P80021	[292-301]	SGCSMGEYFR	-0,064520699	0,07758887
Q3ZD69	[12-25]	SGAQASSTPLSPTR	0	NaN
Q29548	[25-44]	SGAESLGLWPLPFAVDISPR	4,471092088	1,034070988
Q5S3G4	[33-49]	SGGGVPTDEEQATGLER	0,161961121	0,258735945
Q29594	[2-13]	PFSNSHNTLKLR	1,53879945	0,938221076
P05024	[226-238]	SPDFTNENPLETR	1,030282378	0,644587494
P00348	[146-162]	SNTSSLQITSLANATTR	0,030666879	0,010807289
P02554	[138-156]	SLGGGTGSGMGTLLISKIR	1,132456032	0,71973651

Q9N1F5	[8-25]	SLGKGSAPPGPVPEGLIR	-0,346538062	0,128316197
O02772	[23-31]	SIGVGFATR	0,07498618	0,082674872
C0HL13	[2398-2430]	PEDYNVPFTAISVEETAVAVDYD	0	NaN
C0HL13	[2398-2430]	PEDYNVPFTAISVEETAVAVDYD	0	NaN
P08835	[89-105]	SIHTLFGDKLCAIPSLR	1,223370136	0,579206263
Q3ZD69	[51-60]	SLETENAGLR	0,973332261	0,413125428
Q29036	[2-11]	SASVLSVISR	0,105428683	0,191025798
P80928	[2-12]	PMFVVNTNVPR	0,856051391	0,478663437
P80928	[2-12]	PMFVVNTNVPR	0,533244027	0,244364486
P29700	[17-25]	PHGPILGYR	0,022839404	0,007021106
Q29308	[2-16]	PGVTVKDVNQQEFVR	0,538510429	0,402135371
P81045	[1-22]	PGLAAAIPAPPESQEKKPLKPC	-1,088915963	0,812033825
P81045	[1-16]	PGLAAAIPAPPESQEK	0,005025598	0,001980699
O62839	[410-424]	SAPEQTHSALEHCTR	0	NaN
C0HL13	[2335-2362]	EVNYNPCLQNNGGCTHFCFALP	0	NaN
Q1W0Y2	[17-34]	SHYEEGPGKNLPFSVENK	-0,186630798	0,093020945
B1PK17	[169-188]	SIAKYPVGIEVGPQPQGVLR	0,016695661	0,007157651
Q3ZD69	[282-296]	SNLVGAAHEELQQSR	0	NaN
Q6ITQ4	[92-108]	SNEHENAYENTSEEEGR	0	NaN
Q0QF01	[346-352]	SMTLEIR	-1,526648507	1,249709526
C0HL13	[4036-4044]	SMSEHYGER	-0,142980796	0,24950464
P02543	[51-64]	SLYTSSPGGVYATR	0,961031237	0,762316371
P50828	[27-39]	SLTAGPKHGAEGR	-0,245396123	0,303253753
P67937	[6-12]	SLEAVKR	-0,349956211	0,453888165
P50828	[132-152]	SLQEEFPGVPSPLDAAVECHR	0	NaN
Q2XVP4	[187-214]	TTHTTLEHSDCAFMVDNEAIYDI	0	NaN
Q2XVP4	[187-214]	TTHTTLEHSDCAFMVDNEAIYDI	0	NaN
A5GZW8	[59-70]	SKAASLHWTGER	1,424392502	0,835008007
P80147	[230-250]	SKAIHKIDIPSFDWPIAPFPR	0	NaN
O19069	[279-295]	SKPVVSFIAGLTAPPGR	0	NaN
P02543	[87-100]	SLADAINTEFKNTR	1,155999697	0,725498691
Q2EN81	[77-85]	SIMNPYVKR	0,210202236	0,489636898
P00355	[4-11]	VGVNGFGR	1,657157668	0,957579656

A1XQR6	[5-18]	VGPYGQSQPSCFDR	-0,278535726	0,133824426
P01965	[17-31]	VGGQAGAHGAEALER	-0,6588742	0,318902685
P00371	[87-99]	MGLTPVSGYNLFR	1,245533867	0,630682944
P62895	[29-39]	TGPNLHGLFGR	0,057676302	0,059047839
O62839	[445-456]	TFYLNVLNEEER	0	NaN
P79263	[695-707]	SVPDETSHDMDSR	0	NaN
P01025	[670-677]	SVQLMEKR	0,470688544	0,418413899
P00506	[31-53]	SWWAHVEMGPPDPILGVTEAFKI	0	NaN
P79273	[27-42]	TIFQSVELPETYQMLR	0	NaN
Q8MIR4	[394-422]	PGGPAYQPVVEAFGTDILHKDGT	0	NaN
P80928	[75-87]	SYSKLLCGLLAER	1,219516483	0,614385859
Q0QF01	[196-207]	TGHSLLHTLYGR	0	NaN
Q6QAQ1	[52-62]	SYVGDEAQSKR	0,286747232	0,078146523
P50828	[29-39]	TAGPKHGAEGR	1,598821148	0,866267534
P08835	[76-105]	VADESAENCDKSIHTLFGDKLCAIP	0	NaN
C0HL13	[680-688]	TDNGGLGYR	0,134518298	0,18658901
Q6QAQ1	[239-254]	SYELPDGQVITIGNER	0,132426262	0,192387002
Q6QAQ1	[106-116]	TEAPLNPKANR	0,33203709	0,308091917
C0HL13	[3192-3203]	TNIEPYLIFSNR	-0,179905121	0,054001494
P02543	[101-113]	TNEKVELQELNDR	-0,514766642	0,238209227
P05024	[214-225]	SSLTGESEPQTR	0,912837676	0,489181704
P00258	[59-72]	SSSEDKITVHFINR	0,974877634	0,352476272
P09571	[259-280]	SVDGQEDSIWELLNQAQEHFGR	0	NaN
Q29318	[40-55]	SVGICGSDVHYWQHGR	0	NaN
P33198	[301-322]	SVLVCPDGKTIEAEAAHGTVTR	0	NaN
P41367	[25-53]	VPQCEPGSGFSFELTEQQKEFQA	-0,115129084	0,047031976
C0HL13	[4590-4605]	TNFENPIYAETENEPK	0	NaN
P08835	[435-451]	TKKVPQVSTPTLVEVAR	1,068632846	1,009047835
Q9TV61	[1377-1386]	TKYETDAIQR	0,646499979	0,239913786
P33198	[310-322]	TIEAEAAHGTVTR	0	NaN
P08835	[92-105]	TLFGDKLCAIPSLR	1,337492142	0,552622933
P04366	[9-24]	TLPNDIQVQENFDLSR	-1,124690498	0,29617364
P80031	[4-11]	TITYFPVR	2,688958683	1,190794214

Q29099	[109-122]	TMVNYYTSVTPVLR	0	NaN
Q29261	[2-23]	TKLNAQVKGSLNVTTPGVQIWR	0,070294601	0,069470308
A5GZW8	[58-70]	SSKAASLHWTGER	1,284535126	0,883849045
Q70BM6	[2-9]	TFAELVDR	-0,019469836	0,025142829
P80928	[3-12]	MFVVNTNVPR	-1,168368342	0,87384394
Q9N0F1	[69-89]	DDVITVKTPAFAESVTEGDVR	0,233149445	0,354665786
P24854	[23-38]	DEAIHCPPCSEEKLAR	0,434695766	0,524909661
Q9TSX9	[9-22]	DEAPNFEANTTIGR	0,39588743	0,609352699
Q2XVP4	[294-308]	ACFEPANQMVKCDPR	1,138733472	0,697058377
P26044	[490-509]	DENNAEASAELSNDGVMNHR	0	NaN
Q6QAQ1	[4-28]	AALVVDNGSGMCKAGFAGDDAI	-0,375989704	0,180592678
P50441	[224-235]	DQDYPIYSVEDR	0	NaN
Q29092	[22-37]	EDEVDVDGTVEEDLGK	0,26242144	0,792299519
C0HL13	[4485-4499]	DIGVSGFGPESAIDR	0,366812391	0,109661522
P04366	[13-24]	DIQVQENFDLSR	0,402504093	0,120184327
P18648	[71-84]	DNWDSLGSTFTKVR	0,861802887	0,233889487
P01025	[23-35]	DPIYTIITPNVLR	2,149033161	0,87706138
B3SP85	[54-72]	DPPPVNVNLYYESLCNGCR	2,019591306	0,686011957
A1XQS5	[62-99]	FIGAGAATVGVAGSGAGIGTVFG	0	NaN
A1XQS5	[62-68]	DIDTAAK	0,386889053	0,670341239
Q95312	[41-56]	TFASPTQVFFNGANVR	1,140911834	0,652469107
P48819	[20-27]	DQESCKGR	0,895116066	0,323697777
P02067	[95-105]	DQLHVDPENFR	0,566516721	0,650314382
P16276	[634-648]	NAVTQEFGPVPDTAR	0	NaN
Q5S3G4	[31-49]	MASGGGVPTDEEQATGLER	0,811962411	0,768907053
P80021	[335-345]	EAYPGDVFYLH	0	NaN
I3LMB3	[21-37]	EAPQEHDPFTYDYQSLR	1,185226715	0,605897793
Q6QAQ1	[107-116]	EAPLNPKANR	1,949931043	0,957048588
Q9MZ16	[128-139]	DVDFDIAGPSIR	1,397622611	1,18800088
P22412	[17-25]	DQFRDLAVR	-0,303191785	0,406585147
P81405	[2-19]	DVCQDCIQMVTDLQNAVR	1,295192819	1,111901149
P08835	[25-34]	DTYKSEIAHR	0,595173144	0,770956451
P08835	[25-34]	DTYKSEIAHR	0	NaN

Q8HZK3	[1332-1339]	DTLSLHIR	0	NaN
Q9TV61	[1202-1216]	DSVAELGEQIDNLQR	0	NaN
P80021	[197-204]	DSLVPIGR	0,921777163	0,468071288
P06867	[20-38]	DSLDDYVNTQGAFLFSLSR	0	NaN
P81405	[2-19]	DVCQDCIQMVTDLQNAVR	0	NaN
Q3ZD69	[428-435]	SSFSQHAR	0	NaN
Q0QF01	[43-75]	SDAISTQYPVVDHEFDAVVVGAG	0,020642749	0,022514419
F1RKQ4	[9-35]	AGCADDALAGLVACNPSLQLLQG	0	NaN
Q71LE2	[42-50]	YRPGTVALR	-0,002016438	0,002325959
Q2XVP4	[357-373]	YQPPTVVPGGDLAKVQR	0,697604976	0,345391984
Q29545	[261-273];P09571	YLAQVPSHAVVAR	0,845517372	0,344460339
C0HL13	[4551-4584]	NPDELAPDTKPASPSADETQVTKV	0	NaN
Q6QAQ1	[240-254]	YELPDGQVITIGNER	-0,188106733	0,084620914
P06348	[74-82]	YDVEKNNSR	0,329874295	0,501326971
C0HL13	[4632-4647]	YSATEDTFKDTANLVR	-1,144649389	0,460170217
C0HL13	[85-102]	WVCDGEEDCSNGADEHQR	0	NaN
P01965	[14-31]	WGKVGGQAGAHGAEALER	-1,013244767	0,488007588
P18648	[95-106]	WDNLEKETEALR	1,203211021	0,626033883
P02554	[101-121]	WAKGHYTEGAELVDSVLDVVR	-0,428044111	0,221779706
Q29318	[12-20]	VVHGPGDLR	0	NaN
A1E295	[129-141]	VNVEVSAEDMLTC	0	NaN
P29700	[16-25]	VPHGPILGYR	0,473919428	1,110039413
P18648	[22-33]	WQQDDPQSPWDR	0,233149805	0,283383388
Q9MYT8	[2-15]	VPPVQVSPLIKLGR	-0,020079791	0,019812841
Q4U116	[407-438]	IVQMNGDTPHDGGHGGGGHAD	0	NaN
P62936	[2-19]	VNPTVFFDIAVDGEPLGR	0,049986716	0,073971458
P02543	[54-64]	TSSPGGVYATR	-0,104237094	0,101301063
Q6QAQ1	[148-173]	TGIVMDSGDGVTHTVPIYEGYALF	0	NaN
A1XQU1	[44-62]	TTIAGVVYKDGIVLGADTR	0,008792348	0,00901662
Q29554	[111-125]	TTSQEVTQISQEAQR	0	NaN
Q6QAQ1	[297-312]	TVLSGGTTMYPGIADR	0,224911792	0,054216954
Q6Q2J0	[269-291]	VISAIPPTLTAKIHFRPELPSER	0	NaN
Q95332	[89-108]	YVAEKISGQKVNEAACDIAR	1,408503337	0,677534942

Q6QAQ1	[297-312]	TVLSGGTTMYPGIADR	0	NaN
P08835	[26-34]	TYKSEIAHR	0,496316105	0,76910174
P05024	[211-225]	VDNSSLTGESEPQTR	0	NaN
P62802	[61-68]	VFLENVIR	1,428934817	0,808171588
A5A8V7	[22-38]	VFQHGKVEIIANDQGNR	-0,586075968	0,335044899
P02543	[5-12]	TVSSSSYR	-0,312296432	0,142822703
Q28943	[4-21]	VLSKDVADIESILALNPR	0,872896015	0,429074577
P01965	[41-50]	TYFPHFNLSH	0	NaN
P04366	[16-24]	VQENFDLSR	1,113468813	0,427429204
Q4U116	[413-438]	MNGDTPHDGGHGGGGHADCEE	0	NaN
Q4U116	[413-438]	MNGDTPHDGGHGGGGHADCEE	0	NaN
Q02038	[38-45]	SSYTVDGR	0,001667022	0,00230767
P00506	[30-53]	SWWAHVEMGPPDPILGVTEAFK	0,05542099	0,057897181
P00506	[30-53]	SWWAHVEMGPPDPILGVTEAFK	0	NaN
P00506	[30-52]	SSWWAHVEMGPPDPILGVTEAFI	0	NaN
P24964	[64-71]	SSVTHICR	1,0090688	0,429153217
C0HL13	[641-655]	SSTRPFGVTVYHAIR	1,29855801	0,83307521
Q8SQ26	[7-22]	STADGVAVVGSGLIGR	-0,295474035	0,123421487
Q6QAQ1	[232-254]	SSSSLEKSYELPDGQVITIGNER	1,439583813	0,880251079
P01846	[80-105]	SSGFTCQVTHEGTIVEKTVTPSEC	0	NaN
P80147	[29-54]	AAAKVDVEFDYDGPLMKTEVPG	-0,128410493	0,101601811
P10173	[2-7]	SQDSFR	-0,213080367	0,371493548
P16276	[412-424]	SQFTITPGSEQIR	0,234225889	0,637318065
P50441	[270-282]	SQVTNYMGIEWMR	-0,139291997	0,144542192
P15145	[573-599]	AFDYLWIVPISSIKNGVMQDHYW	0	NaN
Q6QAQ1	[233-254]	SSSLEKSYELPDGQVITIGNER	1,353375335	0,770122445
P50441	[38-55]	STQAATASSGNSCAADDK	0	NaN
P50441	[38-55]	STQAATASSGNSCAADDK	0	NaN
P50441	[38-62]	TQAATASSGNSCAADDKATDPLP	0,009214393	0,00631094
P04366	[7-24]	VLTLPNDIQVQENFDLSR	-0,045300932	0,020549182
P00377	[1-28]	LNCIVAVSQNMGIGKNGDLPWP	-0,16566569	0,072146932
Q0QF01	[47-75]	AISTQYPVVDHEFDAVVVGAGGA	0	NaN
P02067	[134-147]	VVAGVANALAHKYH	0	NaN

P29700	[292-304]	VVAVPPGIPPVHR	0,067554941	0,057929969
Q6QAQ1	[9-28]	VVDNGSGMCKAGFAGDDAPR	0,02755706	0,008801893
P20305	[43-62]	VVEHPEFLKAGKEPGLQIWR	-0,088471273	0,033100583
P29700	[287-304]	VVGPMVVAVPPGIPPVHR	0,081520155	0,027254232
Q2EN81	[28-40]	VRPPVQIYGIEGR	1,671001185	1,043316983
P16276	[637-648]	TQEFGPVPDTAR	0,160359729	0,061814085
P00346	[213-229]	TPKVDFPQDQLSTLTGR	-0,329794139	0,158679856
Q29261	[757-788]	SGPLPIFPLEQLVNKPAEELPQGV	0	NaN
Q52NJ6	[97-110]	STYNHLSSWLTDAR	1,486655094	0,89868551
Q767M3	[29-41]	STQSEPHGSPISR	0	NaN
P50441	[38-62]	TQAATASSGNSCAADDKATDPLP	-1,106528286	0,845438882
Q29092	[22-39]	EDEVDVDGTVEEDLGKSR	0,245658024	0,285635697
P06348	[70-82]	AAAGYDVEKNNSR	1,210563575	0,686641271

The difference indicates doxycycline vs. control group. Proteins were considered significant if -log10 (p-value) > 1.3. Significant p-values are marked bold.

Abbreviations: N/A = not available
